# Benefits and harms associated with the use of AI-related algorithmic decision-making systems by healthcare professionals: a systematic review

**DOI:** 10.1016/j.lanepe.2024.101145

**Published:** 2024-12-01

**Authors:** Christoph Wilhelm, Anke Steckelberg, Felix G. Rebitschek

**Affiliations:** a International Graduate Academy (InGrA), Institute of Health and Nursing Science, Medical Faculty, Martin Luther University Halle-Wittenberg, Magdeburger Str. 8, Halle (Saale) 06112, Germany; b Institute of Health and Nursing Science, Medical Faculty, Martin Luther University Halle-Wittenberg, Magdeburger Str. 8, Halle (Saale) 06112, Germany; c Harding Center for Risk Literacy, Faculty of Health Sciences Brandenburg, University of Potsdam, Virchowstr. 2, Potsdam 14482, Germany; d Max Planck Institute for Human Development, Lentzeallee 94, Berlin 14195, Germany

**Keywords:** Algorithmic decision making, ADM, Artificial intelligence, AI, Patient-relevant, Healthcare professionals, Decision support, Benefits, Harms

## Abstract

**Background:**

Despite notable advancements in artificial intelligence (AI) that enable complex systems to perform certain tasks more accurately than medical experts, the impact on patient-relevant outcomes remains uncertain. To address this gap, this systematic review assesses the benefits and harms associated with AI-related algorithmic decision-making (ADM) systems used by healthcare professionals, compared to standard care.

**Methods:**

In accordance with the PRISMA guidelines, we included interventional and observational studies published as peer-reviewed full-text articles that met the following criteria: human patients; interventions involving algorithmic decision-making systems, developed with and/or utilizing machine learning (ML); and outcomes describing patient-relevant benefits and harms that directly affect health and quality of life, such as mortality and morbidity. Studies that did not undergo preregistration, lacked a standard-of-care control, or pertained to systems that assist in the execution of actions (e.g., in robotics) were excluded. We searched MEDLINE, EMBASE, IEEE Xplore, and Google Scholar for studies published in the past decade up to 31 March 2024. We assessed risk of bias using Cochrane's RoB 2 and ROBINS-I tools, and reporting transparency with CONSORT-AI and TRIPOD-AI. Two researchers independently managed the processes and resolved conflicts through discussion. This review has been registered with PROSPERO (CRD42023412156) and the study protocol has been published.

**Findings:**

Out of 2,582 records identified after deduplication, 18 randomized controlled trials (RCTs) and one cohort study met the inclusion criteria, covering specialties such as psychiatry, oncology, and internal medicine. Collectively, the studies included a median of 243 patients (IQR 124–828), with a median of 50.5% female participants (range 12.5–79.0, IQR 43.6–53.6) across intervention and control groups. Four studies were classified as having low risk of bias, seven showed some concerns, and another seven were assessed as having high or serious risk of bias. Reporting transparency varied considerably: six studies showed high compliance, four moderate, and five low compliance with CONSORT-AI or TRIPOD-AI. Twelve studies (63%) reported patient-relevant benefits. Of those with low risk of bias, interventions reduced length of stay in hospital and intensive care unit (10.3 vs. 13.0 days, p = 0.042; 6.3 vs. 8.4 days, p = 0.030), in-hospital mortality (9.0% vs. 21.3%, p = 0.018), and depression symptoms in non-complex cases (45.1% vs. 52.3%, p = 0.03). However, harms were frequently underreported, with only eight studies (42%) documenting adverse events. No study reported an increase in adverse events as a result of the interventions.

**Interpretation:**

The current evidence on AI-related ADM systems provides limited insights into patient-relevant outcomes. Our findings underscore the essential need for rigorous evaluations of clinical benefits, reinforced compliance with methodological standards, and balanced consideration of both benefits and harms to ensure meaningful integration into healthcare practice.

**Funding:**

This study did not receive any funding.

**Research in context**

**Evidence before this study**

Prior to conducting this study, a comprehensive preliminary search of the literature using MEDLINE, EMBASE, IEEE Xplore, and Google Scholar was completed by 29 February 2024. The objective was to identify systematic reviews and meta-analyses that evaluate the patient-relevant outcomes of artificial intelligence (AI)-related algorithmic decision-making (ADM) systems in healthcare. The key search terms employed were ‘artificial intelligence’, ‘machine learning’, ‘medical professionals', decision-making algorithms and outcome-related terms (e.g., adverse events). No previous systematic review has provided an evaluation of the impact of AI-related ADM systems on patient-relevant outcomes. Previous reviews have focused primarily on the diagnostic accuracy and technical performance of AI, with only very limited attention to patient-relevant benefits and harms.

**Added value of this study**

This study provides a comprehensive account of the impact of AI-related ADM systems for health professionals on patient-relevant benefits and harms. The review identifies areas where AI systems have demonstrated potential benefits, such as improvements in depression treatment and pain management. However, it also underscores the inconsistent effects on outcomes like mortality and length of hospital stay. The inclusion of a wide range of healthcare settings and specialties, along with a thorough assessment of bias, points to gaps regarding qualitative AI-related health research. By focusing on preregistered studies and assessing transparency through CONSORT-AI and TRIPOD-AI guidelines, this review offers a more rigorous evaluation than previous studies.

**Implications of all the available evidence**

Available evidence indicates that AI systems have the potential to enhance patient care. The findings of this study underscore the necessity for sustained rigorous assessments of AI-related ADM systems in healthcare. It can be inferred that further research is required to ascertain the full impact on patient-relevant outcomes, particularly in areas such as mortality and quality of life. These insights will be pivotal for shaping future clinical practice, policy decisions, and resource allocation in healthcare.

## Introduction

Advances in artificial intelligence (AI) development enabled complex systems that can outperform medical experts in certain tasks. AI has shown potential to improve diagnostic accuracy,^1^^,^^2^ personalize medicine,^3^ monitor patient care,^4^ and aid drug development.^5^ Among numerous and rapidly growing publications of systematic reviews and meta-analyses on AI in healthcare, many covered breast cancer diagnosis,^6^ ovarian cancer,^7^ skin cancer detection,^8^ COVID-19,^9^ preterm birth prediction,^10^ and diabetes management.^11^ However, it remains uncertain whether these advancements translate into tangible benefits for patients.

Though crucial for AI research and development, primary studies predominantly evaluate the analytical performance of AI systems.^12^, ^13^, ^14^ Performance metrics in terms of accuracy, however, do not imply clinical efficiency,^15^ applicability,^16^ nor improvements in patient care.^17^^,^^18^ Accordingly, Han et al. recently emphasized that while many trials report positive results, they often prioritize diagnostic accuracy over meaningful clinical outcomes.^12^ This contrasts with the substantial rise in approvals of AI respectively machine learning (ML)-based medical devices in the USA and Europe since 2015.^19^ For instance, Plana et al. found that most ML-enabled medical devices approved by the U.S. Food and Drug Administration (FDA) were approved without demonstrated efficacy in randomized controlled trials (RCTs).^20^ These findings point to a critical gap in AI development, when analytical performance is prioritized over patient-relevant outcomes in health care.

In response, this systematic review aims to examine the current state of clinical AI systems’ benefits for patient-relevant outcomes in expert healthcare settings. First, patient-relevant outcomes include benefits and harms such as mortality and morbidity. They reflect the effects of a changing patient health status in response to an intervention. They are distinct from diagnostic outcomes (not due to an intervention) and health status information for health professionals (e.g., physiological states, diagnoses), though being essential for health care.^21^^,^^22^ Second, we focus on AI-related algorithmic decision-making systems (AI-related ADM), while excluding automated decisions by algorithms. We specify AI-related ADM as decision support systems that either apply AI (relying on ML models) or have been developed with the help of AI. Consequently, we review evaluations that compare health professionals’ AI decision support with standard care (usual care). Our work so surpasses recent reviews on healthcare AI.^12^^,^^13^^,^^20^

We only include preregistered clinical trials to counteract publication bias,^23^ minimize selective reporting,^24^ ensure transparency and reproducibility,^25^^,^^26^ and exclude research violating clinical research standards by the World Health Organization and the International Committee of Medical Journal Editors.^27^^,^^28^ Furthermore, we evaluate the quality of research by analyzing studies' risk of bias and transparent reporting of the included studies. Our review ultimately aims to enhance the understanding of AI's role in healthcare, ensuring methodological integrity before innovations are implemented in practice.

## Methods

### Search strategy and selection criteria

In accordance with PRISMA^29^ (Appendix S1 pp 2), we searched MEDLINE and PubMed (via PubMed), EMBASE (via Elsevier), and IEEE Xplore. The search spanned 10 years until March 27, 2024, covering the period when AI-related ADM became relevant in studies. Our search strategy (Appendix S1 pp 8–11) included keywords related to ‘artificial intelligence’, ‘machine learning’, decision-making algorithms, ‘medical professionals', and outcome-related terms, as well as preferred study types. Additional studies were identified by contacting authors of included studies and reviewing reference lists. Grey literature searches were conducted in Google Scholar up to the tenth page.

We included interventional and observational studies published as peer-reviewed full-text articles that met the following inclusion criteria: human patients of any age or sex; the intervention involved an AI decision support system utilizing or developed with ML. Outcomes had to include patient-relevant benefits and harms directly impacting health and quality of life, such as mortality, morbidity (complaints and complications), hospital length of stay, readmission, and health-related quality of life, as defined by the Institute for Quality and Efficiency in Health Care (IQEHC).^21^ We excluded studies without preregistration, without standard of care control, and those outside our scope (e.g., systems supporting the realization of human decisions like robotics).

The study protocol^30^ for this systematic review was pre-registered on PROSPERO (CRD42023412156). Any post-registration amendments and their rationale are documented in the PROSPERO record.

### Data analysis

**Fig. 1:**
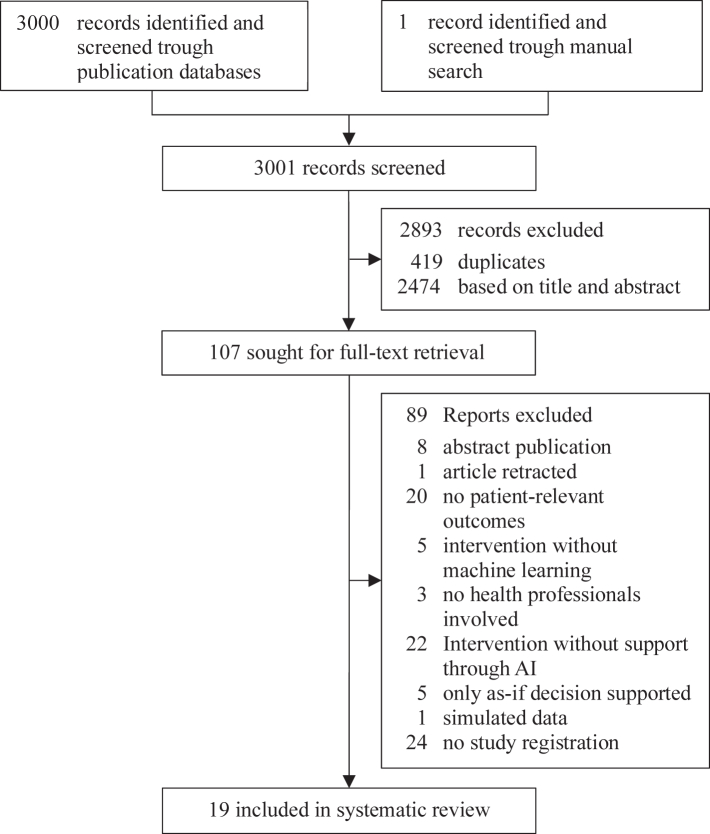
Study selection.

After removing duplicates, two authors (CW and FGR) independently screened all titles and abstracts against the predefined inclusion and exclusion criteria with the help of Rayyan.^29^ They then independently assessed the full texts of potentially eligible studies using Citavi 6^31^ against the same criteria (Fig. 1). Any discrepancies were resolved through discussion. Authors of included studies were contacted if clarification of their data or study methods was required. Using standardized data collection forms, two researchers (CW and FGR) extracted data independently, compared them for discrepancies. Likewise, they evaluated study quality, and reporting. Risk of bias of RCTs was assessed using the revised Cochrane risk-of-bias tool for randomized trials (RoB 2)^32^ and the risk of bias in non-randomized studies for interventions (ROBINS-I) tool.^33^ Transparent reporting of the included studies was assessed trough the Consolidated Standards of Reporting Trials interventions involving Artificial Intelligence (CONSORT-AI) extension^34^ and the Transparent Reporting of a Multivariable Prediction Model for Individual Prognosis Or Diagnosis—Artificial Intelligence (TRIPOD-AI).^35^

Extracted data included country of conduction, setting, study design, observational period, patient-relevant outcomes, intervention, comparator, characteristics of patient and medical professional populations, and characteristics of the used algorithm. Additionally, studies were classified by type of system, medical specialty or clinical area, prediction or classification goal of the AI-related ADM, supported decision, investigated benefits and harms, private or public study funding, applicable regulations [e.g., U.S. Food and Drug Administration (FDA), European Union Medical Device Regulation (EU-MDR)], medical device classification, and commercial availability of the product.

With regard to the heterogeneity in terms of outcome measures, study designs and interventions across studies in the different medical specialties, we present a systematic narrative synthesis of the study characteristics and patient-relevant results.

### Role of the funding source

This research received no specific grant from any funding agency in the public, commercial, or not-for-profit sector.

## Results

Our search retrieved exactly 3,000 records, resulting in 2,581 records after deduplication (Fig. 1). After title and abstract screening 107 records were retained for full-text screening. Of these, 89 were excluded, leaving 18 records after primary screening. One article was identified through a pre-specified manual and citation search in Google Scholar. The result was a total of 19 unique records, 18 RCTs^36^, ^37^, ^38^, ^39^, ^40^, ^41^, ^42^, ^43^, ^44^, ^45^, ^46^, ^47^, ^48^, ^49^, ^50^, ^51^, ^52^, ^53^ and one prospective cohort study,^54^ included in our systematic review. The references and characteristics for all the included studies are available in the appendix (Appendix S2 pp 1). A list of studies excluded during the full-text screening stage, along with brief reasons for exclusion, is also available in the appendix (Appendix S1 pp 12–19).

Of the 19 studies reviewed, most trials were conducted in the USA (9), followed by Europe (4), and China (3). Fourteen studies were conducted in hospitals, three in outpatient settings, one in a nursing home, and one in a mixed setting. Four studies were related to oncology, three to psychiatry, two studies each to internal hospital medicine, neurology, and anesthesiology. Single studies focused on diabetology, long-term care, pulmonology, critical care, audiology, and palliative care.

All study characteristics are detailed in Table 1. Of the 19 studies, six were funded by private and public funds, six by public funds alone, and five by private funds exclusively. One study reported no funding, and another did not disclose funding details.

**Table 1:** Overview over characteristics of 18 RCTs and 1 prospective cohort study (Wang et al. (2021)).

Reference	Country of conduction	Setting	Medical specialty	Intervention system type	Patient-relevant outcome	Funding source
Bailey et al. (2013)^36^	USA	Hospital	Internal hospital medicine	Surveillance system	Intensive care unit transfer, mortality, and length of stay	Private and public
Delgadillo et al. (2022)^37^	UK	Outpatient	Psychiatry	Treatment personalization	Improvement of depression symptoms and anxiety	Private and public
Gong et al. (2020)^38^	China	Hospital	Oncology	Real-time quality improvement system	Adverse events and serious adverse events	Public
Hong et al. (2020)^39^	USA	Hospital	Oncology	Surveillance system	Hospital admission and emergency department admission	Private
Huang et al. (2023)^40^	China	Hospital	Internal hospital medicine	Treatment personalization and reminder system	Incidence of hospital-associated venous thromboembolism, pulmonary embolism, and deep venous thromboembolism	Private and public
Manz et al. (2023)^41^	USA	Hospital	Oncology	Reminder system	Mortality estimates, end-of-life systemic therapy, hospice enrollment, length of stay, inpatient death, and end-of-life intensive care unit admission	Private and public
Martinez-Gutierrez et al. (2023)^42^	USA	Hospital	Neurology	Diagnostic support and surveillance system	Hospital length of stay, mortality, and symptomatic intracerebral hemorrhage	Public
Nimri et al. (2020)^43^	USA, Europe, Israel	Hospital	Diabetology	Surveillance system and treatment personalization	Diabetes-related study adverse events	Private
Park & Moon (2023)^44^	Korea	Nursing home	Long-term care	Surveillance system	Delirium episodes, mortality, length of stay, and 1- and 3-month in-hospital mortality	Public
Pavel et al. (2020)^45^	Ireland, UK, Sweden	Hospital	Neurology	Surveillance system	Inappropriate antiseizure medication	Private and Public
Piette et al. (2022)^46^	USA	Outpatient	Psychiatry	Treatment personalization	Pain-related disability, pain intensity, depression symptoms, health-related quality of life, and patient global impression of change measure	Public
Sadeh-Sharvit et al. (2023)^47^	USA	Hospital and outpatient	Psychiatry	Treatment personalization	Changes in depression, changes in anxiety, and patient satisfaction	Not reported
Schneck et al. (2020)^48^	Germany	Hospital	Anesthesiology	Surveillance system	Length of intensive care unit or Intermediate Care stay (in hours), and length of hospital stay (days)	Private
Seol et al. (2021)^49^	USA	Hospital	Pulmonology	Forecast system and surveillance system	Occurrence of asthma exacerbation within 1-year and adverse events	Private and public
Shimabukuro et al. (2017)^50^	USA	Hospital	Critical care	Surveillance system	Average hospital and intensive care unit length of stay and in-hospital mortality	Public
Wathour et al. (2023)^51^	France	Outpatient	Audiology	Treatment personalization	Hearing performance, fitting comfort, patient satisfaction and adaptation	No funding
Wijnberge et al. (2020)^52^	Netherlands	Hospital	Anesthesiology	Surveillance system	Adverse and serious adverse events	Private
Wilson et al. (2023)^53^	USA	Hospital	Palliative care	Treatment personalization	Hospitalization readmission within 30 days of discharge, Intensive care unit admission, hospital length of stay, 60-day and 90-day hospital readmission	Private
Wang et al. (2021)^54^	China	Hospital	Oncology	Treatment personalization	Chronic pain, quality of life	Public

The studies included a median of 243 patients (IQR 124–828). Eighteen studies reported participant ages, with a median age of 59.3 years (range 0.8–70.6; IQR 41.2–64.0) and two and three studies with children (0–12 years) and seniors (65+ years), respectively. Sex was reported in 18 studies, with a median of 50.5% female participants (range 12.5–79.0; IQR 43.6–53.6). Race or ethnicity was assessed in ten studies, with a median of 71.4% White (non-Hispanic or Latino) participants (range 44.0–95.3; IQR 55.2–88.5).

Twelve studies (63%) reported on the medical professionals who were the intended users, e.g., charge nurses, primary care providers, or endocrinologists. Five of those studies provided user details, e.g., operating endoscopists with one to three years of experience and a total colonoscopy volume of 1500–4000 procedures.^38^ Nine studies informed about user trainings of varying extent: Sadeh-Sharvit et al., for instance, reported a 45-min platform training,^47^ Pavel et al. noted training for monitoring and interpreting electroencephalography (EEG) as well as the algorithm,^45^ Hong et al. (2020) (Hong J, University of California San Francisco, personal communication) reported that physicians were trained on how the model works and how to counsel patients accordingly.^39^ Huang et al. (2023) (Yong L, Shanghai Jiao Tong University School of Medicine, personal communication) embedded the usage of their system and quality control processes within a venous thromboembolism training.^40^ Piette et al. (2022) (Piette JD, University of Michigan, personal communication) reported a two-day training for health professionals with weekly supervision by a chronic pain psychologist.^46^ Seol et al. (2021) (Wi CI, Mayo Foundation for Medical Education and Research, personal communication) noted that primary care providers were trained in person three times on the system, with accompanying materials.^49^ Wathour et al. (2023) (Wathour J, Center d'Audiophonologie, personal communication) reported a weeklong team training on the software use for hearing tests.^51^ Wijnberge et al. (2020) (Wijnberge M, Amsterdam University Medical Center, personal communication) mentioned a demonstration session on how to use the software.^52^

Surveillance systems, used in seven studies, designed to monitor patients in real-time, detect early deterioration, and trigger timely interventions using predictive algorithms and automated alerts, for example, to support the decision whether to transfer a deteriorating patient to the ICU,^36^ or to initiate seizure prevention treatment in neonates.^45^ Systems for treatment personalization, employed in six studies, are designed to tailor medical interventions to the specific needs of each patient, for example, aiding the decision for assigning patients to either low or high-intensity psychiatric care.^37^

Four studies used systems that integrated multiple functionalities, including treatment personalization with reminders,^40^ diagnostic support with surveillance,^42^ combined surveillance with treatment personalization,^43^ and a forecast system paired with surveillance,^49^ For example, continuous blood glucose monitoring in young type 1 diabetes patients provided real-time insulin adjustments to improve glycemic control and reduce complications.^43^

Among the 18 RCTs, four were classified as having low risk of bias, seven showed some concerns, and another seven demonstrated high risk of bias. The most challenging domains identified were measurement of the outcome (10 [56%]) and randomization and intervention assignment (9 [50%]) (Appendix S1 pp 3). The cohort study,^54^ analyzed with ROBINS-I, showed a serious risk of bias, particularly in intervention classification, outcome measurement, and confounding (Appendix S1 pp 4).

Adherence to the CONSORT-AI and TRIPOD-AI checklists (Appendix S1 pp 6–8) varied across the reviewed studies. Of the 18 RCTs analyzed, three showed full compliance, fulfilling 100% of applicable items. Six studies had high compliance (90%–99%), four had moderate compliance (75%–89%), and five had low compliance (less than 75%). The presence of non-applicable items makes direct comparisons difficult, as not all studies were required to address all 37 items. Notably, six out of seven studies published before CONSORT-AI’s release had at least moderate compliance. Among studies published afterwards, four demonstrated high compliance, two moderate but four low compliance. TRIPOD-AI adherence was evaluated in one study, which showed low compliance, as it was published before the introduction of the TRIPOD-AI guidelines. None of the studies cited the CONSORT-AI or TRIPOD-AI guidelines.

Of 19 studies, eleven reported on patient-relevant benefits alone, three reported only harms, and five both. Regarding harms, none of the studies reported an increase in adverse events due to interventions.^38^^,^^40^^,^^43^^,^^45^^,^^47^^,^^49^^,^^50^^,^^52^

Regarding benefits, mortality reduction was subject to five studies. Bailey et al. reported no significant differences in mortality.^36^ Manz et al. found no differences in inpatient death but noted a reduction in systemic therapy at the end of life (OR 0.25, 95% CI: 0.11–0.57, p = 0.001).^41^ In Martinez-Gutierrez et al. the mortality rate declined from 31% in the pre-intervention group to 13% in the post-intervention group (p < 0.001).^42^ And Shimabukuro et al. reported reduced in-hospital mortality (9.0% vs. 21.3%, p = 0.018). Furthermore, subgroup analysis showed that in patients with sepsis, severe sepsis, or septic shock, mortality was reduced from 40.0% in the control group to 13.6% in the intervention group (p = 0.023).^50^

Morbidity outcomes: Delgadillo et al. found that patients with non-complex cases had better depression treatment outcomes in the intervention group (52.3%) compared to the control group (45.1%, p = 0.03).^37^ Sadeh-Sharvit et al. also indicated greater improvement in depression with the Eleos Health Platform (34% improvement, d = 0.82, 95% CI: −0.08–1.66) compared to the control group (20% improvement, d = 0.34, 95% CI: −0.52–1.17).^47^ Both Delgadillo et al. and Piette et al. found no significant differences in anxiety outcomes.^37^^,^^46^ However, Sadeh-Sharvit et al. indicated a larger reduction in anxiety in the intervention group (29% reduction, d = 0.78, 95% CI: −0.11–1.62) than the control group (8% reduction, d = 0.14, 95% CI: −0.70–0.97).^47^ Piette et al. indicated that cognitive behavioral therapy for chronic pain using AI was as effective as standard therapy, with meaningful improvements after six months in pain-related disability (37% vs. 19%, p = 0.01) and pain intensity (29% vs. 17%, p = 0.03) among responders.^46^ Wang et al. showed that predicting beneficial treatments effectively reduced pain scores on the Visual Analog Scale (VAS) (p < 0.05).^54^ Huang et al. noted a lower incidence of venous thromboembolism (OR 0.55, 95% CI: 0.34–0.88, p = 0.01) and deep vein thrombosis (OR 0.50, 95% CI: 0.30–0.83, p = 0.02).^40^

Four studies reported no significant changes in length of hospital stay,^36^^,^^42^^,^^48^^,^^53^ but Shimabukuro et al. found a reduction compared to the control group in both hospital (10.3 vs. 13.0 days, p = 0.042) and ICU lengths of stay (6.3 vs. 8.4 days, p = 0.030).^50^

**Fig. 2:**
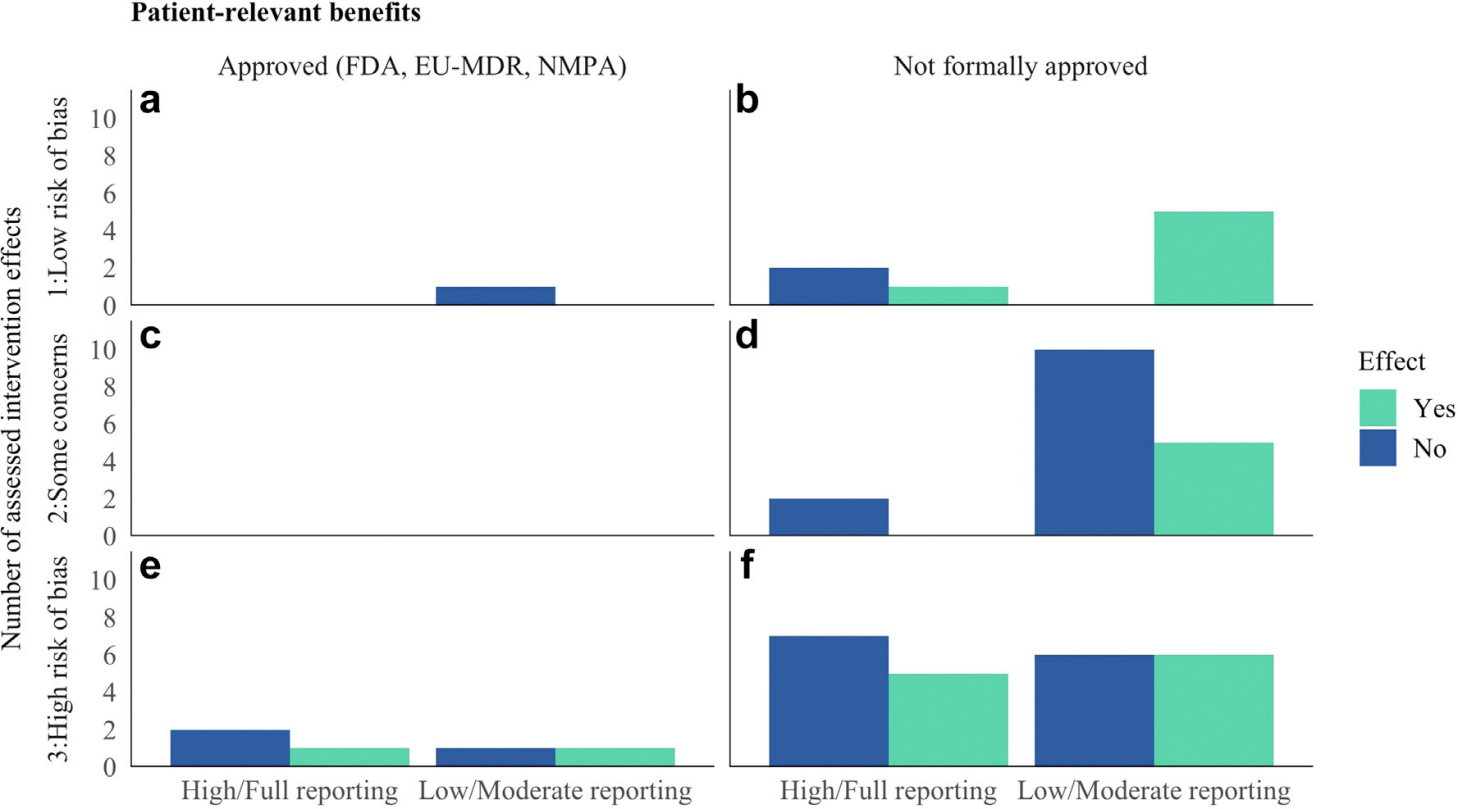
Distribution of intervention effects concerning patient-relevant benefits, according to reporting compliance, risk of bias, and regulatory approval. FDA = U.S. Food and Drug Administration, EU-MDR = European Union Medical Device Regulation, NMPA = National Medical Products Administration (China). Note that most studies are represented by multiple effect assessments.

In Park & Moon, the intervention group had less frequent delirium (p = 0.015) and a higher 1-month in-hospital survival rate (p = 0.026) compared to the control.^44^ Seol et al. found no change in asthma exacerbations within one year (12% vs. 15%, p = 0.626)^49^ Wang et al. reported improved Quality of Life (QoL) with reduced pain and better function, and psychosocial aspects (p < 0.05).^54^ Wathour et al. found that the FOX AI-based fitting system improved speech intelligibility in cochlear implant patients (p < 0.05). But despite better outcomes, 63% still preferred the manual fitting for comfort.^51^ Finally, Wilson et al. found that the AI-CDSS intervention significantly reduced the likelihood of 60-day (OR 0.75, 95% CI: 0.57–0.97) and 90-day (OR 0.72, 95% CI: 0.55–0.93) hospital readmissions, but not for 30 days (OR 0.81, 95% CI: 0.60–1.06).^53^ Overall, effects of AI-related ADM were heterogeneous, also when separated according to reporting compliance with CONSORT-AI, the quality of studies in terms of risk of bias, and regulatory approval (Fig. 2). Regarding the latter, a supplementary search in regulatory bodies’ databases showed that, by August 14, 2024, six of the investigated systems have been approved by national regulatory bodies for health care (FDA, EU-MDR, NMPA). Systematic associations between patient benefits and approval, however, could not be revealed, nor with reporting compliance or study quality.

With regard to preceding development, most algorithms (n = 11) were trained on real-world in-house data (in contrast, n = 4 pooled data sources from different institutions, e.g., neonatal EEG datasets for seizure recognition^45^ and arterial pressure waveforms for hypotension prediction^48^; data sources not specified: n = 4), with large variations in size and types of data sets. For example, Bailey et al.’s development data included heterogeneous patient age, vital-sign data, pharmacy and laboratory information, and ICU transfer data from 28,927 hospital visits.^36^ Similarly, Hong et al. relied on cancer treatment records from 8134 radiotherapy courses including treatment parameters, encounter history, vitals, and laboratory values,^39^ and Piette et al. on patient’s prior levels of physical activity, pain intensity, sleep, CBT skill practice, and session numbers.^46^ Other algorithm developments used less heterogeneous data, stored colonoscopy images and videos from over 5000 patients,^38^ 1167 CT angiography scans,^42^ or arterial waveform data.^52^

The used ML algorithms covered ML model classes broadly, such as gradient boosting (n = 4), neural networks (n = 2), support vector machines (n = 1), random forest models (n = 1), and interpretable models, such as Bayesian classifier (n = 2) and regressions (n = 5).^55^ Five studies omitted respective specifications. Eight out of 19 studies reported about external validation in algorithm development (excluding cross-validation on a given dataset), including three prospective validation studies.

## Discussion

This systematic review is the first that maps patient-relevant benefits and harms that can be attributed to AI-related ADM systems for medical professionals. High-quality studies of patient-relevant outcomes of AI systems in medical decision support are rare—19 we found, compared with media hypes and thousands of AI performance studies. While performance studies can be relatively easily conducted once (simulated) datasets are available, clinical evaluation is effortful, time-consuming, and expensive. The fact that psychiatric and oncological studies were well represented could be an indication of disciplinary priorities in research strategies, data availability, the feasibility of conducting respective clinical trials, the need for decision support, and, in particular, the need for patient-relevant evidence (e.g., for ensuring informed decision-making in cancer screening).^56^ In the future, underrepresented specialties may shift from algorithm performance studies towards patient-relevant evidence in the light of regulatory requirements (e.g., financial compensation). However, recent developments more often enable implementation without causal evidence on benefits of algorithmic technologies,^20^ which is at odds with established requirements to medical devices. As with other medical technologies before, health authorities’ approvals should be tight to patient-related evidence.

The state of evidence can be summarized as follows: While two out of two studies revealed positive effects on depression outcomes and pain, respectively, for other morbidities (1 of 2), mortality (2 of 5), patient anxiety (1 of 3), and the length of stay in hospital (1 of 5) improvements could not be confirmed across most outcomes. Single studies showed positive effects on hospital readmissions and quality of life. This speaks in favor of relevant potential for AI in healthcare. While benefits were consistently revealed in psychiatry (n = 3), and not revealed in anesthesiology (n = 2), the picture was mixed within oncology (n = 4) and neurology (n = 2). However, all interventions have been evaluated over standard care, but not against interventions that can dispense with AI. Future analyses could assure that the AI element is causally relevant for observed benefits.

In addition to this moderate balance as of today, eight studies show a high risk of bias, even after having excluded trials that failed the basic criterion of preregistration. The proportion of 11 out of 19 without high risk indicates that many researchers aim at ensuring high quality in conducting these evaluations. This may contribute to potential implementation strategies. In contrast to that, only five studies provide a balanced benefit-harm assessment of medical professionals’ decision support. Most studies only focus on potential benefits, which is insufficient with regard to informing implementation decisions.^57^ Studies that focus on harms, on the other hand, in most cases lack standardized operationalization in harm assessments, which prevents necessary statements on non-maleficence.^58^ With regard to implementation potentials, both benefits and harms assessments need to meet established standards as known from other health technology research (e.g., cancer screening).

A main issue in patient-relevant AI decision support research is the neglect of the key enabling conditions that have much stronger influence on patient benefits than the algorithms and the data: first, the users who are ought to be supported by the machines. Their capabilities and motivations to interact within their working processes to the benefit of patients depend on trainings and participation.^59^ While half of the studies informed about user trainings—with variant extent between weeklong trainings and less than 1 h—the other half not even described the minimum requirements to an expert user. This makes a successful use less likely—though we could not test this with our work. Introducing ADM for professionals into an existing organizational decision context requires explicit agreements on the roles and preconditions of users and use, respectively. In other words, even highly accurate ML models will fail to be beneficial, if their use is not planned.

Related to that, second, researchers should reveal envisioned implementation conditions with the regard to both error culture—attribution of responsibilities and foreseeable consequences in case of the different types of errors with and without AI decision support—and learning culture.^59^ The latter spells out how errors of the use of such a system are recognized, saved, and subject to communication and other organizational processes, e.g., refinement of use conditions. Requirements to error and learning culture have substantial influence on resulting benefits of an implemented system, but—with a few exceptions—the found studies did not inform about specifications.

Third, who is subject to a system? Can they expect receiving benefits as outlined in the studies? This has two implications, populations, from which evaluation samples have been recruited, reflect validity concerning potential beneficiaries. They have been described in almost all studies. Furthermore, the participation of patients is crucial in developing, piloting, and implementing complex interventions,^59^ such as decision support systems—even for non-emergency and non-surveillance systems. This was rarely reported.

Fourth, opacity hampers future benefits. This not only affects the use of proprietary systems but also the adaptation of data by other researchers, which could result in greater benefits.^60^ Among studies with patient-relevant outcomes, we observe predominant algorithm development on in-house real world data, which underscores potential for more health data collaborations, one rationale for health data spaces.^61^ Independent from patient benefits, we recognize diversity in ML models and development data. In line with prior findings on ML model use,^62^ we cannot recognize that specific model families are more likely to be subject to patient-relevant studies. Noteworthy with regard to transparency, interpretable (linear and Bayesian classifier) and hardly interpretable models (decision tree ensembles, neural networks) were equally represented. Interpretable models have advantages in explaining decisions, enhancing trust, the acceptance and adoption of tools, and empower users.^63^ It need to be noted that the heterogeneous sample of studies on patient-relevant benefits did not allow linking the outcomes to certain types of data and algorithms.

Substantial violations of study reporting standards among the reviewed studies prevent further steps towards implementation, because requirements to a valid system use are not reported, e.g., external validity requirements of patient data sets. A supplementary search in regulatory bodies’ databases (Appendix S1 pp 5) showed that FDA-approved devices for the US were verifiable through the FDA database, but European EU-MDR approvals presented a challenge. The recognition of CE marks under the EU-MDR primarily relied on manufacturer announcements instead of official documents or EUDAMED listings. This is due to the delayed full functionality and non-enforcement of mandatory use of EUDAMED,^64^ limiting access to comprehensive regulatory documentation. This highlights the need for improved transparency and accessibility on approval-relevant information, e.g., for ensuring regulatory oversight.

We recognized furtherly: Many studies focused on short-term outcomes, with limited evaluation of the long-term effects of AI interventions on patient care and health outcomes. Most studies were conducted in high-income countries, potentially limiting the applicability of the findings to lower-income settings or diverse patient populations.

Finally, there were significant gaps in data availability and transparency, with many studies not providing data access or detailed descriptions of the algorithms used and their validations, hindering reproducibility and further research. Benefits of ML models as in ADM depend on their capacity to generalize to unseen data. Our analysis reveals a strong variance in validation practices, from rigorous validation protocols to a lack of reporting on external validation in the most cases. The lack of validation information limits the transferability of findings because the models may not perform as expected outside studied parameters. In addition to that, this raises ethical concerns about the risk of conducting RCTs with patient groups unnecessarily, who have not been subject of prior algorithm development. These limitations highlight the need for more rigorous and comprehensive studies to fully understand the impact of AI systems in healthcare.

Overall, among thousands of studies dedicated to AI health professionals’ decision-making, only five without a high risk of bias studied patient-relevant outcomes, on the basis of an externally validated algorithm—and finally two showed at least one benefit, in depression treatment and with regard to end-of-life care.^37^^,^^41^ This is a delayed beginning and this asymmetric allocation of efforts will change expectably. Nevertheless, it has to be stressed that the desired utility and credibility requires future research to prioritize patient-relevant evidence, particularly generalizable algorithms, study quality and reporting standards.

Our study has several limitations. First, despite an extensive search, only 19 studies met the inclusion criteria, limiting the generalizability of the findings. The included studies varied in their designs, settings, and interventions, making it challenging to draw definitive conclusions across different healthcare contexts. However, our broad scope complements work that focused on individual AI health applications.

Second, our sharp inclusion and exclusion criteria could have created biases. Given the definition of patient-relevant outcomes, for instance, we did not explore the potential of diagnostic AI studies, according to which more accurate diagnoses today may also come with better patient-relevant outcomes in the future. Furthermore, limiting the review to preregistered studies may have excluded potentially relevant studies that were not preregistered, thereby narrowing the scope of the evidence. This approach, however, significantly curbs publication bias and ensures greater transparency and reproducibility, prioritizing methodological rigor over quantity.

Third, our focus omits the benefits of AI in healthcare that are not directly related to patient care. Here are particularly noteworthy the roles of AI for documentation, clinical data collection, system processes, and bio-molecular and preclinical research, e.g., pharmacotherapy development.

Fourth, we used the RoB 2 and ROBINS-I tool at the study level, not on individual outcomes, which could overlook specific biases related to certain outcomes. However, our interpretations might not be strongly affected because specific outcomes could not be compared.

To sum up, for evaluating implementation readiness, future studies of the benefits and harms need to address the benefit-enabling conditions. Our results reiterate the necessity for rigorous methodological standards—adherence to established reporting standards like CONSORT-AI and TRIPOD-AI—and preregistration to ensure validity of AI studies in healthcare. Particularly the lack of detailed training and implementation guidelines for healthcare professionals using AI systems highlights a significant oversight. Crucial bottlenecks are not only input data quality and sufficient external validation but also the conditions of implementation. Furthermore, future studies should examine the human–algorithm interaction, specifically whether medical professionals adhere to the recommendations provided by AI systems and the extent of any deviations. Research shows that compliance with these recommendations can significantly impact outcomes. For example, understanding how and when therapists deviate from a recommender algorithm can provide valuable insights into the effectiveness of AI integration in clinical practice.^65^

Finally, addressing ethical considerations, such as patient consent and data privacy, and promoting interdisciplinary collaboration are essential for the successful use and integration of healthcare data for the development of AI. Ensuring transparency and fostering a learning culture within healthcare organizations will then support the effective implementation of AI technologies. By overcoming these challenges, AI systems can be leveraged to enhance patient care and clinical outcomes significantly. Ultimately, this review emphasizes the importance of a balanced and patient-centered approach in the development and implementation of AI in healthcare.

## Contributors

Christoph Wilhelm (CW) and Dr. Felix G. Rebitschek (FGR) were responsible for the conceptualization and development of the methodology. Christoph Wilhelm led the literature search, data curation, and drafting of the systematic review. He is also the guarantor of the manuscript. Dr. Felix G. Rebitschek contributed to the formal analysis, provided key resources, and oversaw the project administration. He reviewed, edited, and supervised the systematic review process. Prof. Dr. Anke Steckelberg (AS) contributed to the study design and played a critical role in the validation of the methodology. She also reviewed and provided substantial revisions to the manuscript.

All authors had direct access to and verified the underlying data reported in the manuscript. They collectively interpreted the analyses and made significant contributions to the writing, reviewing, and editing of the manuscript.

## Data sharing statement

The data that support the findings of this study are available from the corresponding author, upon reasonable request. Access to the data will be provided to researchers who submit a methodologically sound proposal. The data will be available for 10 years following the article’s publication.

## Declaration of interests

The authors declare no competing interests.

## Appendix A Supplementary data

Appendix S2

Translated Abstract
