# Supplementary material for: Benefits and harms associated with the use of AI-related algorithmic decision-making systems by healthcare professionals: a systematic review
Source: Lancet Reg Health Eur. 2024 Dec 1;48:101145. doi: 10.1016/j.lanepe.2024.101145 (PMC11648885; doi:10.1016/j.lanepe.2024.101145)

# **Table of Contents**

[**Table of Contents 1**](#_Toc175836752)

[**PRISMA 2020 Flow Diagram 2**](#_Toc175836753)

[**Risks of Bias assessment – RoB II 3**](#_Toc175836754)

[**Risks of Bias assessment – ROBINS-I 4**](#_Toc175836755)

[**Regulatory Approval and Commercialization Status of Analyzed Systems 5**](#_Toc175836756)

[**CONSORT-AI Checklist 6**](#_Toc175836757)

[**TRIPOD-AI Checklist 7**](#_Toc175836758)

[**Search Strategies of Databases 9**](#_Toc175836759)

[**List of Excluded Studies with Justifications 12**](#_Toc175836760)

[**PRISMA 2020 Checklist 18**](#_Toc175836761)

# **PRISMA 2020 Flow Diagram**


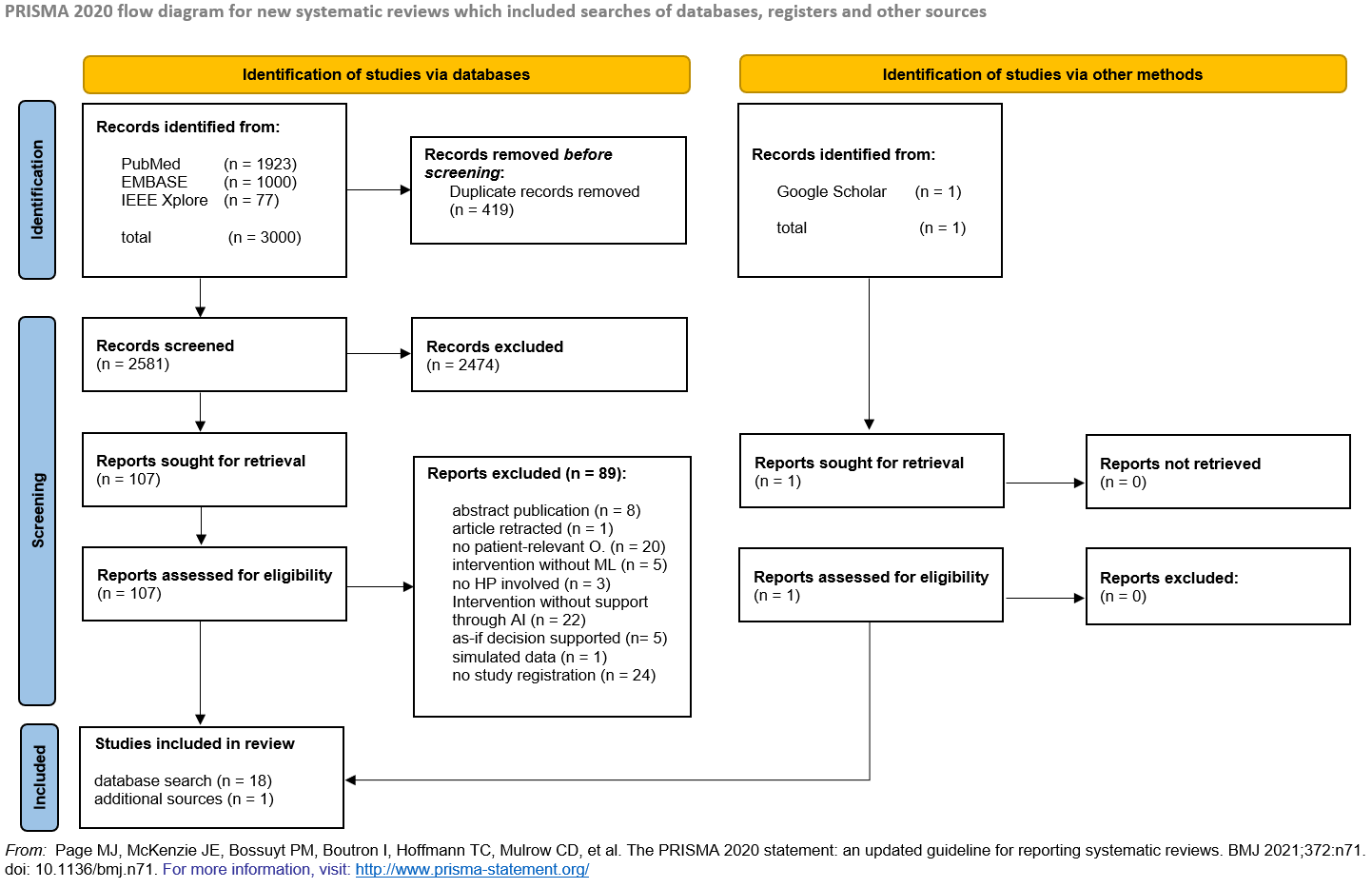


# **Risks of Bias assessment – RoB II**


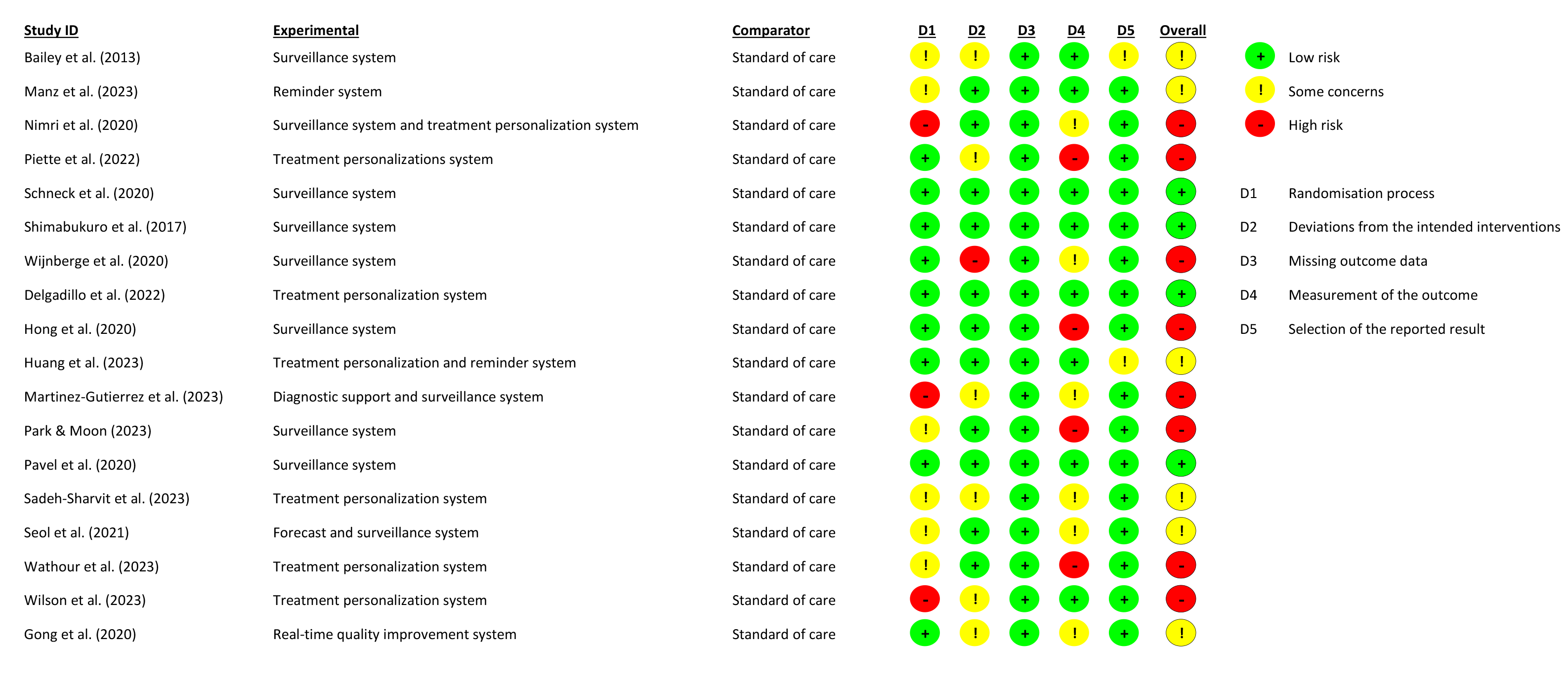


# **Risks of Bias assessment – ROBINS-I**

| **Study ID** | **Experimental** | **Comparator** | **D1** | **D2** | **D3** | **D4** | **D5** | **D6** | **D7** | **Overall** |
| --- | --- | --- | --- | --- | --- | --- | --- | --- | --- | --- |
| Wang et al. (2021) | Treatment personalization system | Standard of care | Serious | Moderate | Serious | Moderate | Moderate | Serious | Moderate | Serious |

D1 Bias due to confounding

D2 Bias in selection of participants into the study

D3 Bias in classification of interventions

D4 Bias due to deviations from intended interventions

D5 Bias due to missing data

D6 Bias in measurement of outcomes

D7 Bias in selection of the reported result

# **Regulatory Approval and Commercialization Status of Analyzed Systems**

Among the 19 systems analyzed, six have been formally approved by regulatory bodies and are commercialized. The system used by Gong et al. is approved by the National Medical Products Administration (NMPA) in China and commercialized by Wuhan ENDOANGEL Medical Technology Co. Ltd.^1^ The system used by Martinez-Gutierrez et al. has FDA approval as a Class II radiological computer-aided triage and notification software^2^, CE marking under EU-MDR, and is commercialized through Viz.ai, Inc.^3^ The System used by Nimri et al. is FDA approved as a Class II insulin therapy adjustment device^4^ also CE-marked, and commercialized by DreaMed Diabetes Ltd^5^. Additionally, the systems in Schneck et al. and Wijnberge et al. have FDA approval as Class II adjunctive predictive cardiovascular indicators later rebranded and commercialized under the Acumen platform by Edwards Lifesciences, including the Acumen IQ finger cuff and Hypotension Prediction Index, both FDA-cleared^6^ and CE-marked^7^. Lastly, the system used by Wathour et al. has CE marking as a Class I device and is commercialized via Cochlear Limited and Otoconsult NV.^8^ The system used by Sadeh-Sharvit et al. lacks formal approval but is widely adopted in U.S. behavioral health organizations to augment clinical workflows^9^.

1. (NMPA) NMPA. Computer Aided Detection Software for Intestinal Polyps in Lower Gastrointestinal Endoscopy Approved for Marketing. 2023. <https://english.nmpa.gov.cn/2023-05/12/c_923528.htm> (accessed August 29 2024).

2. (FDA) USFaDA. 510(k) Premarket Notification: K223042. 2023. <https://www.accessdata.fda.gov/scripts/cdrh/cfdocs/cfPMN/pmn.cfm?ID=K223042> (accessed August 29, 2024).

3. Viz.ai. Viz.ai Receives CE Mark to Bring Life-Saving Stroke Care to Europe. 2023. <https://www.viz.ai/news/viz-ai-receives-ce-mark-to-bring-life-saving-stroke-care-to-europe9> (accessed August 29, 2024).

4. (FDA) USFaDA. 510(k) Premarket Notification: K191370. 2023. <https://www.accessdata.fda.gov/scripts/cdrh/cfdocs/cfPMN/pmn.cfm?ID=K191370> (accessed August 29, 2024).

5. Intelligence SPGM. Article on Trending News: Insulin Therapy Device Gains EU-MDR Approval. 2023. <https://www.spglobal.com/marketintelligence/en/news-insights/trending/5bwdue5kyy_diadgvcpamw2> (accessed August 29, 2024).

6. (FDA) USFaDA. 510(k) Premarket Notification: K183646. 2023. <https://www.accessdata.fda.gov/scripts/cdrh/cfdocs/cfpmn/pmn.cfm?ID=K183646> (accessed August 29, 2024).

7. Lifesciences E. Edwards Launches Acumen Hypotension Prediction Index. 2018. <https://www.edwards.com/newsroom/news/2018-03-19-edwards--acumen-hypotension-prediction-index-launc> (accessed August 29, 2024).

8. Wathour J, Govaerts PJ, Derue L, et al. Prospective Comparison Between Manual and Computer-Assisted (FOX) Cochlear Implant Fitting in Newly Implanted Patients. *Ear Hear* 2023; **44**(3): 494-505.

9. Wellbeing NCfM. Welcoming Eleos Health: A National Council AI Partner. 2023. <https://www.thenationalcouncil.org/eleos-health-partner/> (accessed August 29, 2024).

# **CONSORT-AI Checklist**


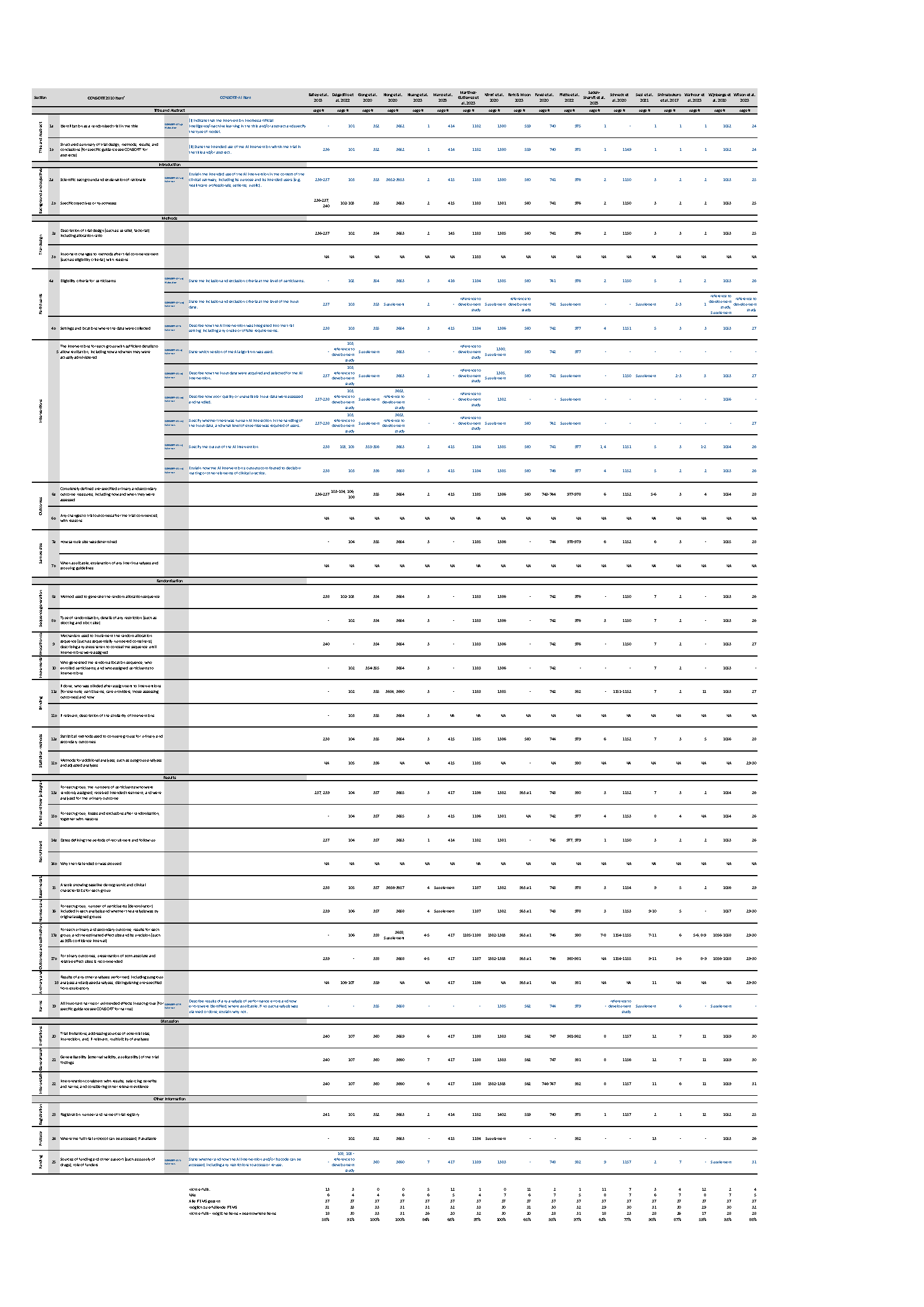


# **TRIPOD-AI Checklist**


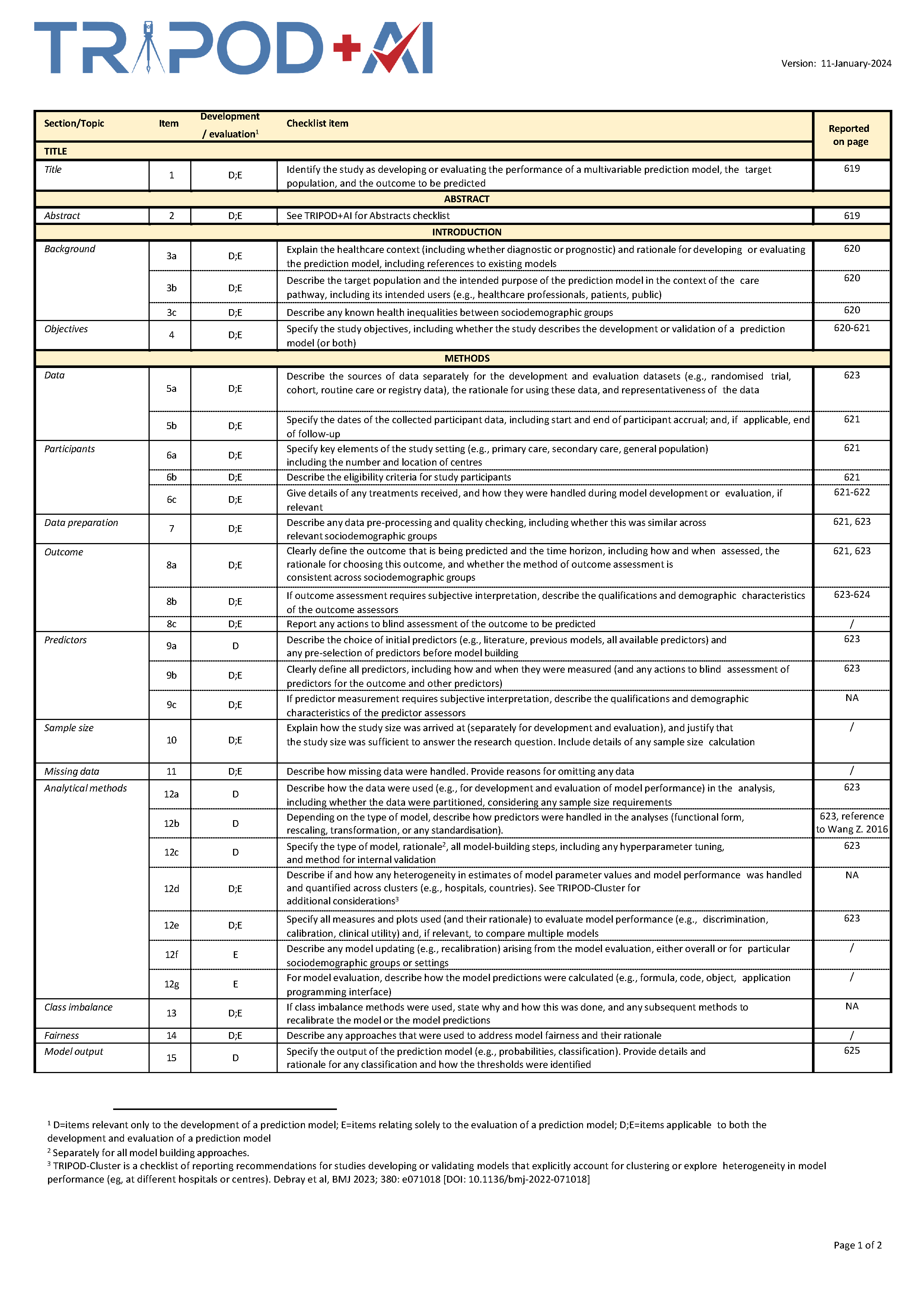


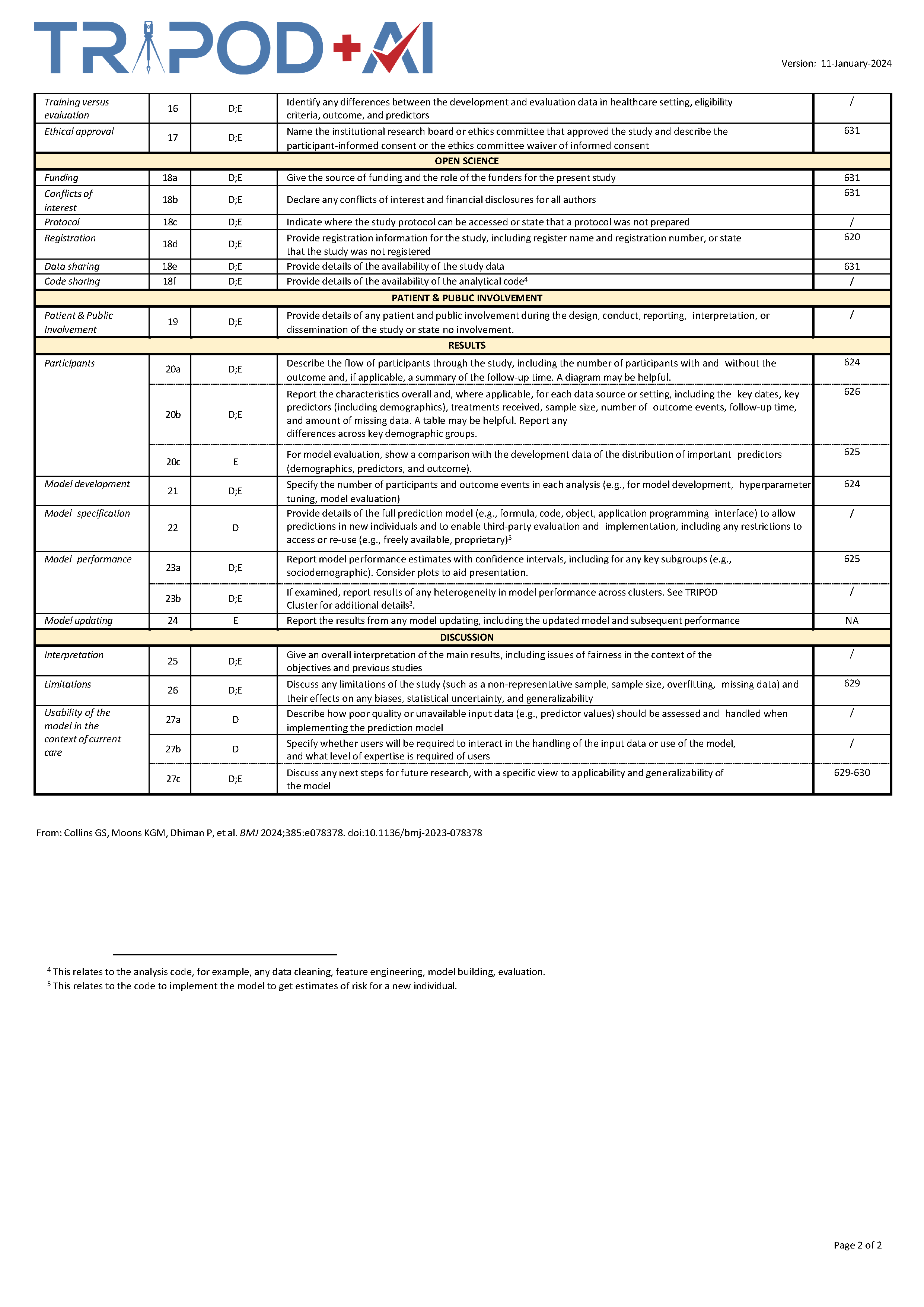


# **Search Strategies of Databases**

**Database: MEDLINE and PubMed via PubMed
Time of search: 27th of March 2024
Results: 1,923 hits**

(("artificial intelligence"[MeSH Terms] OR "artificial intelligence"[Title/Abstract] OR "artificial-intelligence"[Title/Abstract] OR "machine learning"[Title/Abstract] OR "machine-learning"[Title/Abstract] OR "hierarchical learning"[Title/Abstract] OR "computational intelligence"[Title/Abstract] OR "machine intelligence"[Title/Abstract] OR "computer reasoning"[Title/Abstract] OR "deep learning"[Title/Abstract] OR "supervised learning"[Title/Abstract] OR "unsupervised learning"[Title/Abstract] OR "reinforcement learning"[Title/Abstract] OR "representation learning"[Title/Abstract] OR "natural language processing"[Title/Abstract] OR "large language model*"[Title/Abstract] OR "generative model*"[Title/Abstract] OR "representation learning"[Title/Abstract] OR ("knowledge acquisition"[Title/Abstract] AND "computer"[Title/Abstract]) OR ("knowledge representation"[Title/Abstract] AND "computer"[Title/Abstract]) OR "image recognition"[Title/Abstract] OR "machine vision"[Title/Abstract] OR "computer vision"[Title/Abstract] OR "algorithmic decision"[Title/Abstract]))

AND ("expert"[Title/Abstract] OR "experts"[Title/Abstract] OR "medical professional"[Title/Abstract] OR "medical professionals"[Title/Abstract] OR "medical doctor*"[Title/Abstract] OR "physician*"[Title/Abstract] OR "clinician*"[Title/Abstract] OR "general practitioner*"[Title/Abstract] OR "health care professional"[Title/Abstract] OR "health care professionals"[Title/Abstract] OR "healthcare professional"[Title/Abstract] OR "healthcare professionals"[Title/Abstract] OR "nurse"[Title/Abstract] OR "nurses"[Title/Abstract] OR ("therapist"[Title/Abstract] OR "therapists"[Title/Abstract]) OR ("health"[Title/Abstract] AND "alert system"[Title/Abstract]) OR ("medical"[Title/Abstract] AND "alert system"[Title/Abstract]) OR ("practice"[Title/Abstract] AND "alert system"[Title/Abstract]) OR ("hospital"[Title/Abstract] AND "alert system"[Title/Abstract]) OR ("clinic*"[Title/Abstract] AND "alert system"[Title/Abstract]) OR ("health"[Title/Abstract] AND "decision support"[Title/Abstract]) OR ("medical"[Title/Abstract] AND "decision support"[Title/Abstract]) OR ("practice"[Title/Abstract] AND "decision support"[Title/Abstract]) OR ("hospital"[Title/Abstract] AND "decision support"[Title/Abstract]) OR ("clinic*"[Title/Abstract] AND "decision support"[Title/Abstract]) OR ("health"[Title/Abstract] AND "warning system"[Title/Abstract]) OR ("medical"[Title/Abstract] AND "warning system"[Title/Abstract]) OR ("practice"[Title/Abstract] AND "warning system"[Title/Abstract]) OR ("clinic*"[Title/Abstract] AND "warning system"[Title/Abstract]))

AND ("effectiveness"[Title/Abstract] OR "effectivity"[Title/Abstract] OR "benefit"[Title/Abstract] OR "benefits"[Title/Abstract] OR "harm"[Title/Abstract] OR "harms"[Title/Abstract] OR "adverse event*"[Title/Abstract] OR "mortality"[Title/Abstract] OR "morbidity"[Title/Abstract] OR "length of hospital stay"[Title/Abstract] OR "readmission"[Title/Abstract] OR "time to intervention"[Title/Abstract] OR "health-related quality of life"[Title/Abstract] OR "endpoint*"[Title/Abstract] OR "outcome*"[Title/Abstract])

AND ("randomised" OR "randomized" OR "RCT" OR "clinical trial*" OR "cohort" OR "observational study" OR "observational design*" OR "case-control" OR "experiment*" OR "retrospective study" OR "retrospective design*" OR "prospective study" OR "prospective design*" OR "non-inferiority" OR "phase* study" OR "intervention study" OR "diagnostic study" OR "pre-post study" OR "pre post study" OR "pre-post design" OR "pre post design")

AND (y_10[Filter]) AND (humans[Filter])

**Database: Embase via Elsevier
Time of search: 27th of March 2024
Results: 1,000 hits**

('artificial intelligence'/exp OR 'artificial intelligence':ti,ab OR 'artificial-intelligence':ti,ab OR 'machine learning':ti,ab OR 'machine-learning':ti,ab OR 'hierarchical learning':ti,ab OR 'computational intelligence':ti,ab OR 'machine intelligence':ti,ab OR 'computer reasoning':ti,ab OR 'deep learning':ti,ab OR 'supervised learning':ti,ab OR 'unsupervised learning':ti,ab OR 'reinforcement learning':ti,ab OR 'natural language processing':ti,ab OR 'large language model*':ti,ab OR 'generative model*':ti,ab OR 'representation learning':ti,ab OR ('knowledge acquisition':ti,ab AND 'computer':ti,ab) OR ('knowledge representation':ti,ab AND 'computer':ti,ab) OR 'image recognition':ti,ab OR 'machine vision':ti,ab OR 'computer vision':ti,ab OR 'algorithmic decision':ti,ab)

AND ('expert':ti,ab OR 'experts':ti,ab OR 'medical professional':ti,ab OR 'medical professionals':ti,ab OR 'medical doctor*':ti,ab OR 'physician*':ti,ab OR 'clinician*':ti,ab OR 'general practitioner*':ti,ab OR 'health care professional':ti,ab OR 'health care professionals':ti,ab OR 'healthcare professional':ti,ab OR 'healthcare professionals':ti,ab OR 'nurse':ti,ab OR 'nurses':ti,ab OR 'therapist':ti,ab OR 'therapists':ti,ab OR ('health':ti,ab AND 'alert system':ti,ab) OR ('medical':ti,ab AND 'alert system':ti,ab) OR ('practice':ti,ab AND 'alert system':ti,ab) OR ('hospital':ti,ab AND 'alert system':ti,ab) OR ('clinic*':ti,ab AND 'alert system':ti,ab) OR ('health':ti,ab AND 'decision support':ti,ab) OR ('medical':ti,ab AND 'decision support':ti,ab) OR ('practice':ti,ab AND 'decision support':ti,ab) OR ('hospital':ti,ab AND 'decision support':ti,ab) OR ('clinic*':ti,ab AND 'decision support':ti,ab) OR ('health':ti,ab AND 'warning system':ti,ab) OR ('medical':ti,ab AND 'warning system':ti,ab) OR ('practice':ti,ab AND 'warning system':ti,ab) OR ('clinic*':ti,ab AND 'warning system':ti,ab))

AND ('effectiveness':ti,ab OR 'effectivity':ti,ab OR 'benefit':ti,ab OR 'benefits':ti,ab OR 'harm':ti,ab OR 'harms':ti,ab OR 'adverse event*':ti,ab OR 'mortality':ti,ab OR 'morbidity':ti,ab OR 'length of hospital stay':ti,ab OR 'readmission':ti,ab OR 'time to intervention':ti,ab OR 'health-related quality of life':ti,ab OR 'endpoint*':ti,ab OR 'outcome*':ti,ab)

AND ('randomised':ti,ab OR 'randomized':ti,ab OR 'rct':ti,ab OR 'clinical trial*':ti,ab OR 'cohort':ti,ab OR 'observational study':ti,ab OR 'observational design*':ti,ab OR 'case-control':ti,ab OR 'experiment*':ti,ab OR 'retrospective study':ti,ab OR 'retrospective design*':ti,ab OR 'prospective study':ti,ab OR 'prospective design*':ti,ab OR 'non-inferiority':ti,ab OR 'phase* study':ti,ab OR 'intervention study':ti,ab OR 'diagnostic study':ti,ab OR 'pre post study':ti,ab OR 'pre-post study':ti,ab OR 'pre post design':ti,ab OR 'pre-post design':ti,ab)

AND [humans]/lim AND [clinical study]/lim AND [embase]/lim AND [2014-2024]/py AND [article]/lim

**Database: IEEE Xplore via ieeexplore.ieee.org
Time of search: 27th of March 2024
Results: 77 hits**

((("artificial intelligence" OR "artificial-intelligence" OR "machine learning" OR "machine-learning" OR "algorithmic decision" OR "hierarchical learning" OR "computational intelligence" OR "machine intelligence" OR "computer reasoning" OR "deep learning" OR "supervised learning" OR "unsupervised learning" OR "reinforcement learning" OR "representation learning" OR "natural language processing" OR "large language model" OR "large language models" OR "generative models" OR "representation learning" OR "image recognition" OR "machine vision" OR "computer vision")

AND ("length of hospital stay" OR "readmission" OR "time to intervention" OR "health-related quality of life" OR "endpoint" OR "endpoints" OR "outcome" OR "outcomes")

AND ("randomised" OR "randomized" OR "RCT" OR "clinical trial" OR "clinical trials" OR "cohort" OR "observational study" OR "observational design" OR "observational designs" OR "case-control" OR "experiment" OR "experiments" OR "randomised survey" OR "randomized survey" OR "retrospective study" OR "retrospective design" OR "retrospective designs" OR "prospective study" OR "prospective design" OR "prospective designs" OR "non-inferiority" OR "phase study" OR "phases study" OR "intervention study" OR "diagnostic study" OR "pre-post study" OR "pre post study" OR "pre-post design" OR "pre post design")

AND ("expert" OR "experts" OR "medical professional" OR "medical professionals" OR "medical doctor" OR "medical doctors" OR "physician" OR "physicians" OR "clinician" OR "clinicians" OR "general practitioner" OR "health care professional" OR "health care professionals" OR "healthcare professional" OR "healthcare professionals" OR "nurse" OR "nurses" OR "therapist" OR "therapists" OR ("health" AND "warning system") OR ("clinical" AND "decision support") OR ("health" AND "alert system") OR ("medical" AND "alert system") OR ("practice" AND "alert system") OR ("hospital" AND "alert system") OR ("clinical" AND "alert system") OR ("health" AND "decision support") OR ("medical" AND "decision support") OR ("practice" AND "decision support") OR ("hospital" AND "decision support") OR ("medical" AND "warning system") OR ("practice" AND "warning system") OR ("clinical" AND "warning system"))

AND ("effectiveness" OR "effectivity" OR "benefit" OR "benefits" OR "harm" OR "harms" OR "adverse event" OR "adverse events" OR "mortality" OR "morbidity" OR "length of hospital stay" OR "readmission" OR "time to intervention" OR "health-related quality of life" OR "endpoint" OR "endpoints" OR "outcome" OR "outcomes")))

Filters Applied: Journals, Early Access Articles, Magazines, Publication Date 2014 – 2024

# **List of Excluded Studies with Justifications**

**Abstract Publication/ Authors Where Contacted – No Answer or No Following Publication (n= 8)**

Barton C, Mohamadlou H, Lynn-Palevsky A, Fletcher G, Shieh L, Stark P, Chettipally U, Shimabukuro DW, Feldman M, Das R. Predicting patient mortality: using machine learning to identify at-risk patients and improve outcomes. Am J Respir Crit Care Med. 2018;197. Available from: https://www.cochranelibrary.com/central/doi/10.1002/central/CN-01620837/full

Cheng C-L, Kuo Y-L, Liu N-J, Lien J-M, Tang C-P, Hsieh Y-H, Leung FW. Comparing Left Colon Mucus Production by Water versus Saline Infusion During Water Exchange Colonoscopy: a Prospective Randomized Controlled Trial. Am J Gastroenterol. 2022;117(10). Available from: https://doi.org/10.14309/01.ajg.0000859004.36866.dd

Liu X. Evaluating the impact of an integrated computer-based decision support with person-centered analytics for the management of hypertension: a randomized controlled trial. J Hypertens. 2018;36. Available from: https://doi.org/10.1097/01.hjh.0000547994.70348.44

Parikh RB, Zhang Y, Small D, Chivers C, Evans CN, Regli SB, Braun J, Hanson CW, Bekelman JE, Gabriel PE, et al. Long-term effect of machine learning-triggered behavioral nudges on serious illness communication and end-of-life outcomes among patients with cancer: a randomized clinical trial. J Clin Oncol. 2022;40(16). Available from: https://doi.org/10.1200/JCO.2022.40.16

Pelah A, Sarangi V, de Villiers E, Shenker N, Stone T, Estibeiro P, Barenholtz E, Levy X, Fields G. An artificial intelligence platform for movement analysis and rehabilitation: clinical applications of stepsense to complex pain and long covid. Physiotherapy. 2022;114‐e75. Available from: https://doi.org/10.1016/j.physio.2021.12.011

Schwab K. Point-of-care AI-driven ER to EP referrals for non-valvular atrial fibrillation patients in the emergency department. Cardiovasc Digit Health J. 2023;4(5). Available from: https://doi.org/10.1016/j.cvdhj.2023.08.004

Shamanna P, Joshi S, Shah L, Dharmalingam M, Vadavi A, Damodaran S, Mohammed J, Mohamed M, Poon T, Keshavamurthy A, et al. Remission of T2DM by digital twin technology with reduction of cardiovascular risk: interim results of randomised controlled clinical trial. Eur Heart J. 2022;43‐i223. Available from: https://doi.org/10.1093/eurheartj/ehab849.177

Faqar-Uz-Zaman SF, Anantharajah L, Baumartz P, Sobotta P, Filmann N, Zmuc D, Wagner M von, Detemble C, Sliwinski S, Marschall U, Bechstein WO, Schnitzbauer AA. The Diagnostic Efficacy of an App-based Diagnostic Health Care Application in the Emergency Room: Eradar-trial. A prospective, Double-blinded, Observational Study. Ann Surg. 2022;276(5):935–942. Available from: https://doi.org/10.1097/SLA.0000000000005614

**Article has Been Retracted (n= 1)**

Ni S, Li X, Yi X. Clinical Application of Artificial Intelligence: Auto-Discerning the Effectiveness of Lidocaine Concentration Levels in Osteosarcoma Femoral Tumor Segment Resection: THIS ARTICLE HAS BEEN RETRACTED. J Healthc Eng. 2022;2022:7069348. Available from: https://doi.org/10.1155/2022/7069348

**No Patient-relevant Outcomes (n=20)**

Abramoff MD, Whitestone N, Patnaik JL, Rich E, Ahmed M, Husain L, Hassan MY, Tanjil MS, Weitzman D, Dai T, et al. Autonomous artificial intelligence increases real-world specialist clinic productivity in a cluster-randomized trial. Npj Digit Med. 2023;6(1):184. Available from: https://doi.org/10.1038/s41746-023-00931-7

Clifton DA, Wong D, Clifton L, Wilson S, Way R, Pullinger R, Tarassenko L. A large-scale clinical validation of an integrated monitoring system in the emergency department. IEEE J Biomed Health Inform. 2013;17(4):835–842. Available from: https://doi.org/10.1109/JBHI.2012.2234130

Fang C, Pan Y, Zhao L, Niu Z, Guo Q, Zhao B. A machine learning-based approach to predict prognosis and length of hospital stay in adults and children with traumatic brain injury: retrospective cohort study. J Med Internet Res. 2022;24(12). Available from: https://doi.org/10.2196/41819

He F, Page JH, Weinberg KR, Mishra A. The development and validation of simplified machine learning algorithms to predict prognosis of hospitalized patients with COVID-19: multicenter, retrospective study. J Med Internet Res. 2022;24(1). Available from: https://doi.org/10.2196/31549

Koutsouleris N, Kambeitz-Ilankovic L, Ruhrmann S, Rosen M, Ruef A, Dwyer DB, Paolini M, Chisholm K, Kambeitz J, Haidl T, et al. Prediction models of functional outcomes for individuals in the clinical high-risk state for psychosis or with recent-onset depression: a multimodal, multisite machine learning analysis. JAMA Psychiatry. 2018;75(11):1156–1172. Available from: https://doi.org/10.1001/jamapsychiatry.2018.2165

Liu P, Wang P, Glissen Brown JR, Berzin TM, Zhou G, Liu W, Xiao X, Chen Z, Zhang Z, Zhou C, et al. The single-monitor trial: an embedded CADe system increased adenoma detection during colonoscopy: a prospective randomized study. Ther Adv Gastroenterol. 2020;13. Available from: https://doi.org/10.1177/1756284820979165

Liu W-C, Lin C, Lin C-S, Tsai M-C, Chen S-J, Tsai S-H, Lin W-S, Lee C-C, Tsao T-P, Cheng C-C. An artificial intelligence-based alarm strategy facilitates management of acute myocardial infarction. J Pers Med. 2021;11(11). Available from: https://doi.org/10.3390/jpm11111149

Maeda Y, Kudo S-E, Ogata N, Misawa M, Iacucci M, Homma M, Nemoto T, Takishima K, Mochida K, Miyachi H, et al. Evaluation in real-time use of artificial intelligence during colonoscopy to predict relapse of ulcerative colitis: a prospective study. Gastrointest Endosc. 2022;95(4):747-756.e2. Available from: https://doi.org/10.1016/j.gie.2021.10.019

Manz C, Parikh RB, Evans CN, Chivers C, Regli SB, Changolkar S, Bekelman JE, Small D, Rareshide CA, O’Connor N, et al. Effect of integrating machine learning mortality estimates with behavioral nudges to increase serious illness conversions among patients with cancer: a stepped-wedge cluster randomized trial. J Clin Oncol. 2020;38(15). Available from: https://doi.org/10.1200/JCO.2020.38.15_

Mišić VV, Rajaram K, Gabel E. A simulation-based evaluation of machine learning models for clinical decision support: application and analysis using hospital readmission. Npj Digit Med. 2021;4(1). Available from: https://doi.org/10.1038/s41746-021-00468-7

Narang A, Bae R, Hong H, Thomas Y, Surette S, Cadieu C, Chaudhry A, Martin R, McCarthy PM, Rubenson DS, Goldstein S, Little SH, Lang RM, Weissman NJ, Thomas JD. Utility of a deep-learning algorithm to guide novices to acquire echocardiograms for limited diagnostic use. JAMA Cardiol. 2021;6(6):624–632. Available from: https://doi.org/10.1001/jamacardio.2021.0185

Ng K, Kartoun U, Stavropoulos H, Zambrano JA, Tang PC. Personalized treatment options for chronic diseases using precision cohort analytics. Sci Rep. 2021;11(1):1139. Available from: https://doi.org/10.1038/s41598-021-80967-5

Nicolae C, Semple M, Luc L, Smith M, Chung HT, Loblaw A, Morton G, Mendez LC, Tseng C-L, Davidson M, et al. Results of a phase I randomized-controlled trial evaluating conventional vs. machine learning treatment planning for prostate low-dose-rate brachytherapy. Brachytherapy. 2018;17(4)‐S60. Available from: https://www.cochranelibrary.com/central/doi/10.1002/central/CN-01620066/full

Repici A, Spadaccini M, Antonelli G, Correale L, Maselli R, Galtieri PA, Pellegatta G, Capogreco A, Milluzzo SM, Lollo G, et al. Artificial intelligence and colonoscopy experience: lessons from two randomised trials. Gut. 2022;71(4):757–765. Available from: https://doi.org/10.1136/gutjnl-2021-324471

Rushlow DR, Croghan IT, Inselman JW, Thacher TD, Friedman PA, Yao X, Pellikka PA, Lopez-Jimenez F, Bernard ME, Barry BA, Attia IZ, Misra A, Foss R, Molling PE, Rosas SL, Noseworthy PA. Clinician adoption of an artificial intelligence algorithm to detect left ventricular systolic dysfunction in primary care. Mayo Clin Proc. 2022;97(11):2076–2085. Available from: https://doi.org/10.1016/j.mayocp.2022.04.008

Sher DJ, Godley A, Park Y, Carpenter C, Nash M, Hesami H, Zhong X, Lin M-H. Prospective study of artificial intelligence-based decision support to improve head and neck radiotherapy plan quality. Clin Transl Radiat Oncol. 2021;29:65–70. Available from: https://doi.org/10.1016/j.ctro.2021.05.006

Wang P, Liu X, Berzin TM, Glissen Brown JR, Liu P, Zhou C, Lei L, Li L, Guo Z, Lei S, et al. Effect of a deep-learning computer-aided detection system on adenoma detection during colonoscopy (CADe-DB trial): a double-blind randomised study. Lancet Gastroenterol Hepatol. 2020;5(4):343-351. Available from: https://doi.org/10.1016/S2468-1253(19)30411-X

Wang P, Liu X-G, Kang M, Peng X, Shu M-L, Zhou G-Y, Liu P-X, Xiong F, Deng M-M, Xia H-F, et al. Artificial intelligence empowers the second-observer strategy for colonoscopy: a randomized clinical trial. Gastroenterol Rep. 2023;11. Available from: https://doi.org/10.1093/gastro/goac081

Wu L, Zhang J, Zhou W, An P, Shen L, Liu J, Jiang X, Huang X, Mu G, Wan X, Lv X, et al. Randomised controlled trial of WISENSE, a real-time quality improving system for monitoring blind spots during esophagogastroduodenoscopy. Gut. 2019;68(12):2161-2169. Available from: https://doi.org/10.1136/gutjnl-2018-317366

Yao L, Li X, Wu Z, Wang J, Luo C, Chen B, Luo R, Zhang L, Zhang C, Tan X, et al. Effect of artificial intelligence on novice-performed colonoscopy: a multicenter randomized controlled tandem study. Gastrointest Endosc. 2024;99(1):91-99.e9. Available from: https://doi.org/10.1016/j.gie.2023.07.044

**intervention Without Machine Learning (n = 5)**

Bie AJR de, Mestrom E, Compagner W, Nan S, van Genugten L, Dellimore K, Eerden J, van Leeuwen S, van de Pol H, Schuling F, Lu X, Bindels A, Bouwman ARA, Korsten EHHM. Intelligent checklists improve checklist compliance in the intensive care unit: a prospective before-and-after mixed-method study. Br J Anaesth. 2021;126(2):404–414. Available from: https://doi.org/10.1016/j.bja.2020.09.044

Bull LM, Arendarczyk B, Reis S, Nguyen A, Werr J, Lovegrove-Bacon T, Stone M, Sherlaw-Johnson C. Impact on all-cause mortality of a case prediction and prevention intervention designed to reduce secondary care utilisation: findings from a randomised controlled trial. Emerg Med J. 2023. Available from: https://doi.org/10.1136/emermed-2022-212908

Elliott LS, Henderson JC, Neradilek MB, Moyer NA, Ashcraft KC, Thirumaran RK. Clinical impact of pharmacogenetic profiling with a clinical decision support tool in polypharmacy home health patients: a prospective pilot randomized controlled trial. PLoS One. 2017;12(2). Available from: https://doi.org/10.1371/journal.pone.0170905

Kimmel SE, French B, Kasner SE, Johnson JA, Anderson JL, Gage BF, Rosenberg YD, Eby CS, Madigan RA, McBane RB, et al. A pharmacogenetic versus a clinical algorithm for warfarin dosing. N Engl J Med. 2013;369(24):2283–2293. Available from: https://doi.org/10.1056/NEJMoa1310669

Xing F, Guo Y, Xia N, Zhang S, Yin J, Qin L, Zhu C, Gao Q, Jia J, Zhao Y, Qi Y, Li W. Mobile APP-assisted family physician program for improving blood pressure outcome in hypertensive patients. BMC Prim Care. 2023;24(1):8. Available from: https://doi.org/10.1186/s12875-023-01965-2

**intervention Without Health Professionals (n= 3)**

Jiao T, Zhang Y, Liou T, Stevens V, Young D, Brixner D. The optimal treatment regime to delay the onset of mucoid pseudomonas aeruginosa pulmonary infection on pediatric cystic fibrosis patients. Cochrane Database Syst Rev. 2017;20(5). Available from: https://www.cochranelibrary.com/central/doi/10.1002/central/CN-01407770/full

Lo L-J, Yang C-T, Ho C-T, Liao C-H, Lin H-H. Automatic assessment of 3-dimensional facial soft tissue symmetry before and after orthognathic surgery using a machine learning model: a preliminary experience. Ann Plast Surg. 2021;86(3). Available from: https://doi.org/10.1097/SAP.0000000000002687

Strømskov S, Weinel RA, Ghaderi A, Andersson H, Parmskog N, Hjort E, Wärn AS, Jannert M, Andersson G. Effects of tailored and ACT-influenced internet-based CBT for eating disorders and the relation between knowledge acquisition and outcome: a randomized controlled trial. Behav Ther. 2017;48(5):624–637. Available from: https://doi.org/10.1016/j.beth.2017.02.002

**Intervention Without Support of Health Professionals Through AI (n = 22)**

Chen S, Jiang L, Zhang E, Hu S, Wang T, Gao F, Zhang N, Wang X, Zheng J. A novel nomogram based on machine learning-pathomics signature and neutrophil to lymphocyte ratio for survival prediction of bladder cancer patients. Front Oncol. 2021;11. Available from: https://doi.org/10.3389/fonc.2021.703033

Chen Y, Wu S, Ye J, Wu M, Xiao Z, Ni X, Wang B, Chen C, Tan X, Liu R. Predicting all-cause mortality risk in atrial fibrillation patients: a novel LASSO-Cox model generated from a prospective dataset. Front Cardiovasc Med. 2021;8. Available from: https://doi.org/10.3389/fcvm.2021.730453

Ito-Masui A, Sakamoto R, Matsuo E, Kawamoto E, Motomura E, Tanii H, Yu H, Sano A, Imai H, Shimaoka M. Effect of an internet-delivered cognitive behavioral therapy-based sleep improvement app for shift workers at high risk of sleep disorder: single-arm, nonrandomized trial. J Med Internet Res. 2023;25. Available from: https://doi.org/10.2196/45834

Kim D, Hwang JE, Cho Y, Cho HW, Lee W, Lee JH, Oh IY, Baek S, Lee E, Kim J. A retrospective clinical evaluation of an artificial intelligence screening method for early detection of STEMI in the emergency department. J Korean Med Sci. 2022;37(10). Available from: https://doi.org/10.3346/jkms.2022.37.e81

Komorowski M, Celi LA, Badawi O, Gordon AC, Faisal AA. The Artificial Intelligence Clinician learns optimal treatment strategies for sepsis in intensive care. Nat Med. 2018;24(11):1716–1720. Available from: https://doi.org/10.1038/s41591-018-0213-5

Kwong J, Khondker A, Tran C, Evans E, Cozma AI, Javidan A, Ali A, Jamal M, Short T, Papanikolaou F, et al. Explainable artificial intelligence to predict the risk of side-specific extraprostatic extension in pre-prostatectomy patients. Can Urol Assoc J. 2022;16(6). Available from: https://doi.org/10.5489/cuaj.7473

Lin R, Stanley MD, Ghassemi MM, Nemati S. A deep deterministic policy gradient approach to medication dosing and surveillance in the ICU. Conf Proc IEEE Eng Med Biol Soc. 2018;2018:4927–4931. Available from: https://doi.org/10.1109/EMBC.2018.8513203

Lu Y, Pareek A, Wilbur RR, Lel DP, Krych AJ, Camp CL. Understanding anterior shoulder instability through machine learning: new models that predict recurrence, progression to surgery, and development of arthritis. Orthop J Sports Med. 2021;9(11). Available from: https://doi.org/10.1177/23259671211053326

Okada Y, Matsuyama T, Morita S, Ehara N, Miyamae N, Jo T, Sumida Y, Okada N, Watanabe M, Nozawa M, et al. Machine learning-based prediction models for accidental hypothermia patients. J Intensive Care. 2021;9(1). Available from: https://doi.org/10.1186/s40560-021-00525-z

Olano-Espinosa E, Avila-Tomas JF, Minue-Lorenzo C, Matilla-Pardo B, Serrano Serrano ME, Martinez-Suberviola FJ, Gil-Conesa M, Del Cura-González I. Effectiveness of a conversational chatbot (Dejal@bot) for the adult population to quit smoking: pragmatic, multicenter, controlled, randomized clinical trial in primary care. JMIR Mhealth Uhealth. 2022;10(6). Available from: https://doi.org/10.2196/34273

Pan J, Wu G, Yu J, Geng D, Zhang J, Wang Y. Detecting the early infarct core on non-contrast CT images with a deep learning residual network. J Stroke Cerebrovasc Dis. 2021;30(6):105752. Available from: https://doi.org/10.1016/j.jstrokecerebrovasdis.2021.105752

Qiao N, Ma Y, Chen X, Ye Z, Ye H, Zhang Z, Wang Y, Lu Z, Wang Z, Xiao Y, Zhao Y. Machine learning prediction of visual outcome after surgical decompression of sellar region tumors. J Pers Med. 2022;12(2). Available from: https://doi.org/10.3390/jpm12020152

Radosavljevic V, Ristovski K, Obradovic Z. A data-driven acute inflammation therapy. BMC Med Genomics. 2013;6. Available from: https://doi.org/10.1186/1755-8794-6-S3-S7

Shao J, Liu F, Ji S, Song C, Ma Y, Shen M, Sun Y, Zhu S, Guo Y, Liu B, et al. Development, external validation, and visualization of machine learning models for predicting occurrence of acute kidney injury after cardiac surgery. Rev Cardiovasc Med. 2023;24(8). Available from: https://doi.org/10.31083/j.rcm2408229

Wen R, Wang M, Bian W, Zhu H, Xiao Y, He Q, Wang Y, Liu X, Shi Y, Hong Z, Xu B. Machine learning-based prediction of symptomatic intracerebral hemorrhage after intravenous thrombolysis for stroke: a large multicenter study. Front Neurol. 2023;14. Available from: https://doi.org/10.3389/fneur.2023.1247492

Wu W, Zhou Z. A comprehensive way to access hospital death prediction model for acute mesenteric ischemia: a combination of traditional statistics and machine learning. Int J Gen Med. 2021;14:591–602. Available from: https://doi.org/10.2147/IJGM.S300492

Yang L, Dong X, Abuduaini B, Jiamali N, Seyiti Z, Shan XF, Gao XM. Development and validation of a nomogram to predict mortality risk in patients with ischemic heart disease. Front Cardiovasc Med. 2023;10. Available from: https://doi.org/10.3389/fcvm.2023.1115463

Yang S, Cao L, Zhou Y, Hu C. A retrospective cohort study: predicting 90-day mortality for ICU trauma patients with a machine learning algorithm using XGBoost using MIMIC-III database. J Multidiscip Healthc. 2023;16:2625–2640. Available from: https://doi.org/10.2147/JMDH.S416943

Zhang G, Yang H, Zhu X, Luo J, Zheng J, Xu Y, Zheng Y, Wei Y, Mei Z, Shao G. A CT-based radiomics nomogram to predict complete ablation of pulmonary malignancy: a multicenter study. Front Oncol. 2022;12. Available from: https://doi.org/10.3389/fonc.2022.841678

Zhang Q, Wang J, Liu G, Zhang W. Artificial intelligence can use physiological parameters to optimize treatment strategies and predict clinical deterioration of sepsis in ICU. Physiol Meas. 2023;44(1). Available from: https://doi.org/10.1088/1361-6579/acb03b

Zheng H, Ryzhov IO, Xie W, Zhong J. Personalized multimorbidity management for patients with type 2 diabetes using reinforcement learning of electronic health records. Drugs. 2021;81(4):471–482. Available from: https://doi.org/10.1007/s40265-020-01435-4

Zou W, Wang Z, Wang F, Zhang G, Liu R. A nomogram predicting overall survival in patients with non-metastatic pancreatic head adenocarcinoma after surgery: a population-based study. BMC Cancer. 2021;21(1):524. Available from: <https://doi.org/10.1186/s12885-021-08250-4>

**As-if Decision Supported (n=5)**

Bowness JS, Burckett-St Laurent D, Hernández N, Keane PA, Lobo C, Margetts S, Moka E, Pawa A, Rosenblatt M, Sleep N, Taylor A, Woodworth G, Vasalauskaite A, Noble JA, Higham H. Assistive artificial intelligence for ultrasound image interpretation in regional anaesthesia: an external validation study. Br J Anaesth. 2023;130(2):217–225. Available from: https://doi.org/10.1016/j.bja.2022.06.031

Greene JJ, Tavares J, Guarin DL, Hadlock T. Clinician and automated assessments of facial function following eyelid weight placement. JAMA Facial Plast Surg. 2019;21(5):387–392. Available from: https://doi.org/10.1001/jamafacial.2019.0086

Petch J, Nelson W, Wu M, Ghassemi M, Benz A, Fatemi M, Di S, Carnicelli A, Granger C, Giugliano R, Hong H, Patel M, Wallentin L, Eikelboom J, Connolly SJ. Optimizing warfarin dosing for patients with atrial fibrillation using machine learning. Sci Rep. 2024;14(1):4516. Available from: https://doi.org/10.1038/s41598-024-55110-9

Shi J, Ding X, Liu X, Li Y, Liang W, Wu J. Automatic clinical target volume delineation for cervical cancer in CT images using deep learning. Med Phys. 2021;48(7):3968–3981. Available from: https://doi.org/10.1002/mp.14898

Xiao Y, Zhang J, Chi C, Ma Y, Song A. Criticality and clinical department prediction of ED patients using machine learning based on heterogeneous medical data. Comput Biol Med. 2023;165:107390. Available from: https://doi.org/10.1016/j.compbiomed.2023.107390

**Fully Simulated/ No Real World Data (n = 1)**

Tardini E, Zhang X, Canahuate G, Wentzel A, Mohamed ASR, van Dijk L, Fuller CD, Marai GE. Optimal treatment selection in sequential systemic and locoregional therapy of oropharyngeal squamous carcinomas: deep Q-learning with a patient-physician digital twin dyad. J Med Internet Res. 2022;24(4). Available from: https://doi.org/10.2196/29455

**No Study Registration (n= 24)**

Adams R, Henry KE, Sridharan A, Soleimani H, Zhan A, Rawat N, Johnson L, Hager DN, Cosgrove SE, Markowski A, et al. Prospective, multi-site study of patient outcomes after implementation of the TREWS machine learning-based early warning system for sepsis. Nat Med. 2022;28(7):1455–1460. Available from: https://doi.org/10.1038/s41591-022-01894-0

Barakat-Johnson M, Jones A, Burger M, Leong T, Frotjold A, Ratcliff S, Fethney J, Coyer F. Reshaping wound care: evaluation of an artificial intelligence app to improve wound assessment and management. Stud Health Technol Inform. 2024;310:941–945. Available from: https://doi.org/10.3233/SHTI231103

Bassin L, Raubenheimer J, Bell D. The implementation of a real-time early warning system using machine learning in an Australian hospital to improve patient outcomes. Resuscitation. 2023;188. Available from: https://doi.org/10.1016/j.resuscitation.2023.109821

Boussina A, Shashikumar SP, Malhotra A, Owens RL, El-Kareh R, Longhurst CA, Quintero K, Donahue A, Chan TC, Nemati S, Wardi G. Impact of a deep learning sepsis prediction model on quality of care and survival. Npj Digit Med. 2024;7(1). Available from: https://doi.org/10.1038/s41746-023-00986-6

Burns J, Williams D, Mlinaritsch D, Koechlin M, Canning T, Neitzel A. Early detection and treatment of acute illness in medical patients with novel software: a prospective quality improvement initiative. BMJ Open Qual. 2022;11(3). Available from: https://doi.org/10.1136/bmjoq-2022-001845

Byvaltsev V, Kalinin А. Assessment of clinical decision support system efficiency in spinal neurosurgery for personalized minimally invasive technologies used on lumbar spine. Sovrem Tehnol Med. 2021;13(5):13–21. Available from: https://doi.org/10.17691/STM2021.13.5.02

Chi S, Kim S, Reuter M, Ponzillo K, Oliver DP, Foraker R, Heard K, Liu J, Pitzer K, White P, Moore N. Advanced care planning for hospitalized patients following clinician notification of patient mortality by a machine learning algorithm. JAMA Netw Open. 2023;6(4). Available from: https://doi.org/10.1001/jamanetworkopen.2023.8795

Fox SR, Toomu A, Gu K, Kang J, Sung K, Han FT, Hoffmayer KS, Hsu JC, Raissi F, Feld GK, et al. Impact of artificial intelligence arrhythmia mapping on time to first ablation, procedure duration, and fluoroscopy use. J Cardiovasc Electrophysiol. 2024. Available from: https://doi.org/10.1111/jce.16237

Hinson JS, Klein E, Smith A, Toerper M, Dungarani T, Hager D, Hill P, Kelen G, Niforatos JD, Stephens RS, et al. Multisite implementation of a workflow-integrated machine learning system to optimize COVID-19 hospital admission decisions. Npj Digit Med. 2022;5(1). Available from: https://doi.org/10.1038/s41746-022-00646-1

Kessler S, Desai M, McConnell W, Jai EM, Mebine P, Nguyen J, Kiroyan C, Ho D, Schweber E von, Schweber L von. Economic and utilization outcomes of medication management at a large Medicaid plan with disease management pharmacists using a novel artificial intelligence platform from 2018 to 2019: a retrospective observational study using regression methods. J Manag Care Spec Pharm. 2021;27(9):1186–1196. Available from: https://doi.org/10.18553/jmcp.2021.21036

Liu W, Yu X, Wang J, Zhou T, Yu T, Chen X, Xie S, Han F, Wang Z. Improving kidney outcomes in patients with nondiabetic chronic kidney disease through an artificial intelligence-based health coaching mobile app: retrospective cohort study. JMIR Mhealth Uhealth. 2023;11. Available from: https://doi.org/10.2196/45531

Nellis JR, Sun Z, Chang B, Della Porta G, Mantyh CR. A risk-prediction platform for acute kidney injury and 30-day readmission after colorectal surgery. J Surg Res. 2023;292:91–96. Available from: https://doi.org/10.1016/j.jss.2023.07.040

Ni Y, Lingren T, Hall ES, Leonard M, Melton K, Kirkendall ES. Designing and evaluating an automated system for real-time medication administration error detection in a neonatal intensive care unit. J Am Med Inform Assoc. 2018;25(5):555–563. Available from: https://doi.org/10.1093/jamia/ocx156

Rösler ÁM, Fraportti J, Nectoux P, Constantin G, Cazella S, Nunes MRP, Lucchese F, Pardo AO. Development and application of a system based on artificial intelligence for transcatheter aortic prosthesis selection. Braz J Cardiovasc Surg. 2018;33(4):391–397. Available from: https://doi.org/10.21470/1678-9741-2018-0072

Schmuelling L, Franzeck FC, Nickel CH, Mansella G, Bingisser R, Schmidt N, Stieltjes B, Bremerich J, Sauter AW, Weikert T, Sommer G. Deep learning-based automated detection of pulmonary embolism on CT pulmonary angiograms: no significant effects on report communication times and patient turnaround in the emergency department nine months after technical implementation. Eur J Radiol. 2021;141:109816. Available from: https://doi.org/10.1016/j.ejrad.2021.109816

Segal G, Segev A, Brom A, Lifshitz Y, Wasserstrum Y, Zimlichman E. Reducing drug prescription errors and adverse drug events by application of a probabilistic, machine-learning based clinical decision support system in an inpatient setting. J Am Med Inform Assoc. 2019;26(12):1560–1565. Available from: https://doi.org/10.1093/jamia/ocz135

Shamanna P, Joshi S, Shah L, Dharmalingam M, Saboo B, Mohammed J, Mohamed M, Poon T, Kleinman N, Thajudeen M, Keshavamurthy A. Type 2 diabetes reversal with digital twin technology-enabled precision nutrition and staging of reversal: a retrospective cohort study. Clin Diabetes Endocrinol. 2021;7(1). Available from: https://doi.org/10.1186/s40842-021-00134-7

Shamanna P, Saboo B, Damodharan S, Mohammed J, Mohamed M, Poon T, Kleinman N, Thajudeen M. Reducing HbA1c in type 2 diabetes using digital twin technology-enabled precision nutrition: a retrospective analysis. Diabetes Ther. 2020;11(11):2703–2714. Available from: https://doi.org/10.1007/s13300-020-00931-w

Solares GJ, Garcia D, Monge Garcia MI, Crespo C, Rabago JL, Iglesias F, Larraz E, Zubizarreta I, Rabanal JM. Real-world outcomes of the hypotension prediction index in the management of intraoperative hypotension during non-cardiac surgery: a retrospective clinical study. J Clin Monit Comput. 2023;37(1):211–220. Available from: https://doi.org/10.1007/s10877-022-00881-7

Winkler JK, Blum A, Kommoss K, Enk A, Toberer F, Rosenberger A, Haenssle HA. Assessment of diagnostic performance of dermatologists cooperating with a convolutional neural network in a prospective clinical study: human with machine. JAMA Dermatol. 2023;159(6):621–627. Available from: https://doi.org/10.1001/jamadermatol.2023.0905

Winslow CJ, Edelson DP, Churpek MM, Taneja M, Shah NS, Datta A, Wang CH, Ravichandran U, McNulty P, Kharasch M, Halasyamani LK. The impact of a machine learning early warning score on hospital mortality: a multicenter clinical intervention trial. Crit Care Med. 2022;50(9):1339–1347. Available from: https://doi.org/10.1097/CCM.0000000000005492

Yadav S, Sethi R, Pradhan A, Vishwakarma P, Bhargari M, Gattani R, Chaturvedi S, Chaudhary G, Sharma A, Dwivedi SK, et al. ‘Routine’ versus ‘smart phone application based ‐ intense’ follow up of patients with acute coronary syndrome undergoing percutaneous coronary intervention: impact on clinical outcomes and patient satisfaction. Int J Cardiol Heart Vasc. 2021;35. Available from: https://doi.org/10.1016/j.ijcha.2021.100832

Yi M, Lin Y, Lin Z, Xu Z, Li L, Huang R, Huang W, Wang N, Zuo Y, Li N, Ni D, Zhang Y, Li Y. Biopsy or follow-up: AI improves the clinical strategy of US BI-RADS 4A breast nodules using a convolutional neural network. Clin Breast Cancer. 2024. Available from: https://doi.org/10.1016/j.clbc.2024.02.003

Zhou S, Ma X, Jiang S, Huang X, You Y, Shang H, Lu Y. A retrospective study on the effectiveness of artificial intelligence-based clinical decision support system (AI-CDSS) to improve the incidence of hospital-related venous thromboembolism (VTE). Ann Transl Med. 2021;9(6). Available from: https://doi.org/10.21037/atm-21-1093

# **PRISMA 2020 Checklist**


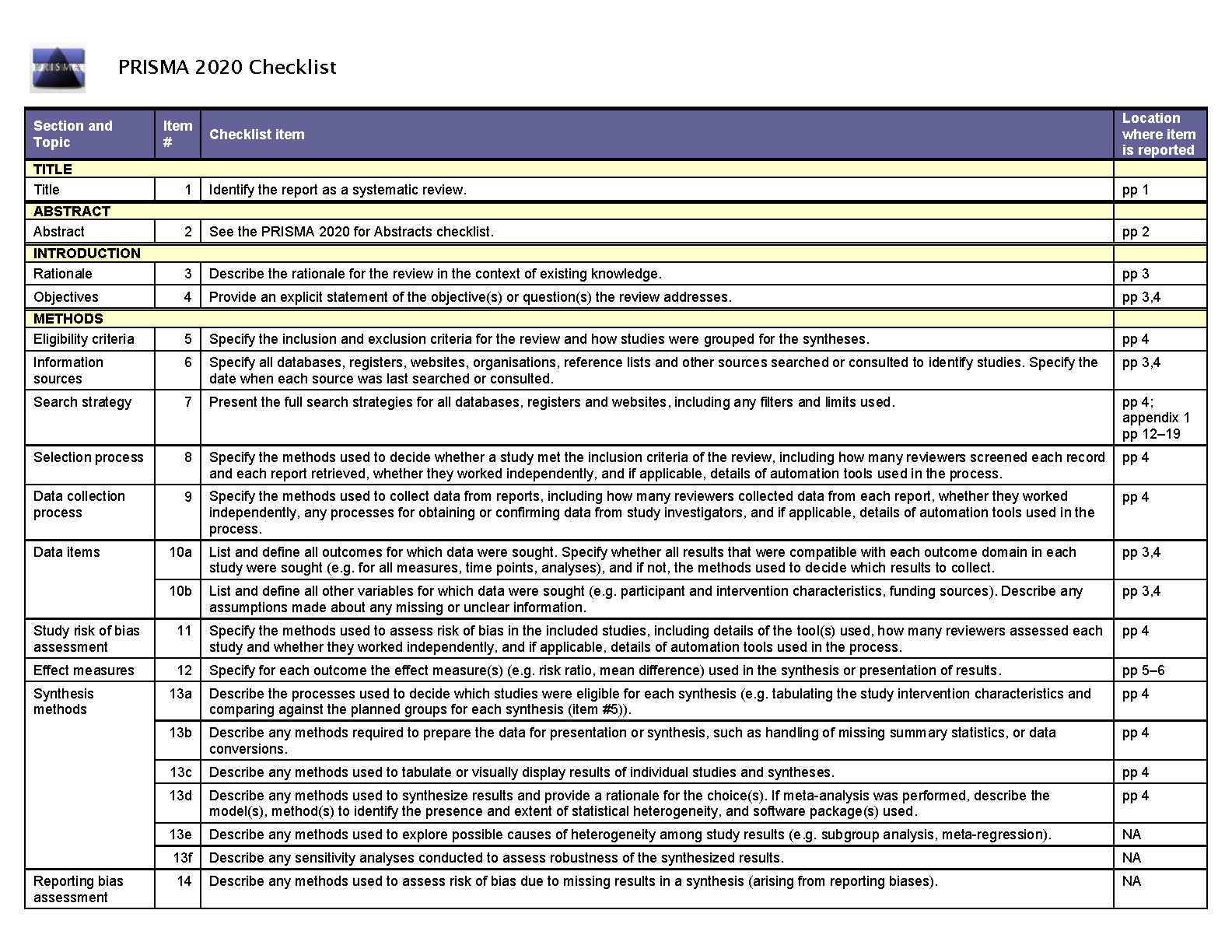


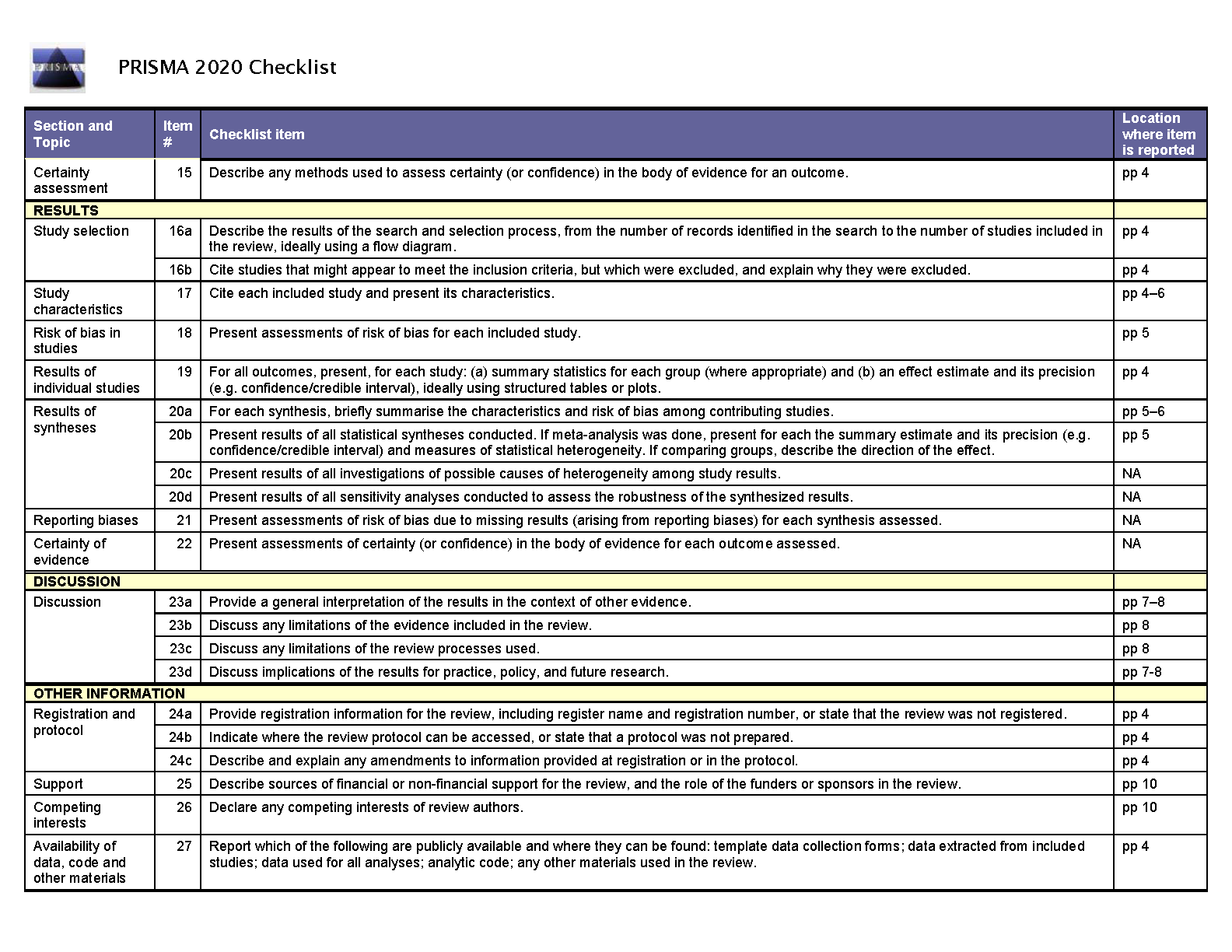

Supplement: Appendix S1 [file mmc1.docx]
