# Supplementary material for: Benefits and harms associated with the use of AI-related algorithmic decision-making systems by healthcare professionals: a systematic review
Source: Lancet Reg Health Eur. 2024 Dec 1;48:101145. doi: 10.1016/j.lanepe.2024.101145 (PMC11648885; doi:10.1016/j.lanepe.2024.101145)
Supplement: Translated Abstract [file mmc3.docx]

*Editors’ disclaimer: This translation in German was submitted by the authors and we reproduce it as supplied. It has not been peer reviewed. Our editorial processes have only been applied to the original abstract in English, which should serve as reference for this manuscript.*

**Zusammenfassung**

**Hintergrund**

Trotz bedeutender Fortschritte im Bereich der Künstlichen Intelligenz (KI), die es komplexen Systemen ermöglichen, einzelne Aufgaben akkurater als medizinische Fachkräfte zu erfüllen, bleibt der Einfluss auf patientenrelevante Ergebnisse ungewiss. Um diese Lücke zu schließen, bewertet diese systematische Übersichtsarbeit Nutzen und Schäden, die mit dem Einsatz von KI-gestützten algorithmischen Entscheidungssystemen (AI-ADM), die von Gesundheitsfachkräften genutzt werden, verbunden sind, im Vergleich zur Standardversorgung.

**Methoden**

In Übereinstimmung mit den PRISMA-Richtlinien wurden Interventions- und Beobachtungsstudien eingeschlossen, die als peer-reviewte Volltextartikel veröffentlicht wurden und die folgenden Kriterien erfüllten: menschliche Patienten; Interventionen, die algorithmische Entscheidungssysteme beinhalten, die mit und/oder unter Verwendung von maschinellem Lernen (ML) entwickelt wurden; und Ergebnisse, die patientenrelevante Nutzen und Schäden beschreiben, welche die Gesundheit und Lebensqualität direkt betreffen, wie beispielsweise Mortalität und Morbidität. Studien, die nicht präregistriert wurden, keine Kontrollgruppe mit Standardversorgung beinhalteten oder sich auf Systeme bezogen, welche die Ausführung von Handlungen unterstützen (z.B. in der Robotik), wurden ausgeschlossen. Wir durchsuchten MEDLINE, EMBASE, IEEE Xplore und Google Scholar nach Studien, die in den letzten zehn Jahren bis zum 31. März 2024 veröffentlicht wurden. Das Verzerrungsrisiko wurde unter Verwendung der Werkzeuge RoB-2 und ROBINS-I von Cochrane bewertet, die Transparenz im Reporting mit Hilfe von CONSORT-AI und TRIPOD-AI. Zwei Forscher führten die Prozesse unabhängig voneinander durch und lösten Konflikte durch Diskussionen. Diese Übersichtsarbeit wurde bei PROSPERO registriert (CRD42023412156) und das Studienprotokoll wurde veröffentlicht.

**Ergebnisse**

Von 2.582 Studien erfüllten 18 randomisierte kontrollierte Studien (RCTs) und eine Kohortenstudie die Einschlusskriterien. Sie repräsentierten Fachgebiete wie Psychiatrie, Onkologie und Innere Medizin. Insgesamt umfassten die Studien im Mittel etwa 243 Patienten (IQR 124–828) mit einem Median-Anteil von 50,5% Teilnehmerinnen (Spanne 12,5–79,0%, IQR 43,6–53,6%). Bei vier Studien wurde das Verzerrungsrisiko als gering, bei sieben mit gewissen Bedenken und bei weiteren sieben als hoch oder schwerwiegend eingestuft. Die Transparenz der Berichterstattung variierte erheblich: sechs Studien zeigten eine hohe, vier eine moderate und fünf eine niedrige Übereinstimmung mit den CONSORT-AI- bzw. TRIPOD-AI-Richtlinien. Zwölf Studien (63 %) berichteten einen patientenrelevanten Nutzen. In den Studien mit niedrigem Verzerrungsrisiko reduzierten die Interventionen gegenüber den Kontrollgruppen die Aufenthaltsdauer im Krankenhaus und auf der Intensivstation (10,3 vs. 13,0 Tage, p=0,042; 6,3 vs. 8,4 Tage, p=0,030), die innerklinische Mortalität (9,0% vs. 21,3%, p=0,018) sowie Depressionssymptome bei nicht-komplexen Fällen (45,1% vs. 52,3%, p=0,03). Allerdings gaben nur acht Studien (42%) über Schäden wie unerwünschte Ereignisse Auskunft, wobei keine von keiner Zunahme durch die Interventionen berichtete.

**Interpretation**

Die derzeitige Evidenz zu KI-gestützten ADM-Systemen liefert nur begrenzte Erkenntnisse über patientenrelevanten Nutzen und Schäden. Unsere Ergebnisse unterstreichen den dringenden Bedarf an rigorosen Einschätzungen möglicher klinischer Vorteile, nach der verstärkten Einhaltung methodischer Standards und einer ausgewogenen Betrachtung sowohl von Nutzen als auch von Schäden, um eine sinnvolle Integration in die Gesundheitsversorgung sicherzustellen.

**Finanzierung**

Diese Studie erhielt keine finanzielle Unterstützung.
